# Supplementary material for: Circulation of West Nile virus in mosquitoes approximate to the migratory bird stopover in West Coast Malaysia
Source: PLoS Negl Trop Dis. 2023 Apr 6;17(4):e0011255. doi: 10.1371/journal.pntd.0011255 (PMC10112790; doi:10.1371/journal.pntd.0011255)
Supplement: S1 Table — (DOCX) [file pntd.0011255.s001.docx]

**Supplementary Table 1.** Partial DNA Sequence of WNV RNA from Mosquitoes

| ID sequence | Type of Primer | DNA Nucleotide sequences |
| --- | --- | --- |
| UPM59-Perak | Forward | GTGGATTGCGATGCAGCTCTAGTGAGTCTCAGACTGAACTAATGCTTCTGACTGCGCCATCAACCGGCGGAGCTCAAAACAAAAACA |
|  | Reverse | GTGGATTGCGATGCAGCTCTAGTGAGTCTCAGACTGAACTAATGCTTCTGACTGCGCCATCAACCGGCGGAGCTCAAAACAAAAACA |
| UPM5-Selangor | Forward | GAAGAAGCATCTCTTGAGTTTCAAGAAAGAACTAGGAACCCTGACCAGCGCCATCAACCGGCGGAGCTCAAAACAAAAAGT |
|  | Reverse | CTGCATTGCGCTGCAGCTCTAGTGAGTTCAGACAGACTAGGAACCCTGACCAGCGCCATCAACCGGCGGAGCTCTAAACAAAAACAGTTGTTTTTTTTTTGTGGGGGTGTTTTCTTTATTTATGTTTTTGATTTTGTTATTTCCTCCCCCTTTGGGTTTTTT |
| UPM14-Selangor | Forward | CCCTTTCCAAATTGCGATGAGCATCTCTTGAGTTTCAGAAGAACTAGGAACCCTGACCAGCGCCATCAACCGGCGGAGCTCAAAACAAAAA |
|  | Reverse | TCGCTCATGCCGATGAGCTCTAGTGAGTCTCAGACAGAACTAGGAACCCTGACCAGCGCCATCAACCGGCGGATCTCTAAACTAAAACA |
| UPM17-Selangor | Forward | TGCAGTTTCACGAAAGAACTAGGAACCCTGACCAGCGCCATCAACCGGCGGAGCTCAAAACAAAAAAGTTGTGTGTTCCCCTCCGGGAGCGTGGGGTTGCGACCGCC |
|  | Reverse | ACGGCCGAGACTTATCGAGCTCTACTTAGTGTCTCGGACACAACTAGGAACCCTGACCAGCGCCATCAACCGGCGGAGCTCAAAACAAAAACAAATGTTCGCG |
| UPM23-Selangor | Forward | GAGAAGCTCTCTTGAGTTTCAAGAAAGAACTAGGAACCCTGGACCAGCGCCATCAACCGGCGGAGCTCAAAACAAAAA |
|  | Reverse | ACGGAATAGCGAGGGGCTCTAGTGAGTCTCGGACAGAACAGATGCTCCCTGACCAGCGCGATCGACCGGCGGATCTCTAAAATAAAACA |
| UPM38-Selangor | Forward | CCACCTACCGCGGCGACGACGCTCTTCTTGAGTTTCAAGAAAGAACTAGGAACCCTGACCAGCGCCATCAACCGGCGGAGCTCAAAACAAAAAA |
|  | Reverse | AGACTACTACGTAGAGTCTTAGGACACAACAGTGCTCCCTGACTGCGCCATCAACCGGCGGAGCTCAAAACAAAAACATCGAT |
| UPM47-Selangor | Forward | CCCAAATCTGGGATGAGCATCTCTTGAGTTCAGAAGACTAGGAACCCTGACCAGCGCCATCAACCGGCGGAGCTCAAAACAAAAA |
|  | Reverse | ACGGGCGTTGGCGAAGGAGCTCTAGTGAGTCTCGGACAGAACAGATGCTTCTGACTGCGCGATCGACCAGCGGATCTCTCGATCCAACA |
| UPM49-Selangor | Forward | AAAAAAACAAGAGGTATTCGGAGCAAGCCATCTCTTGCGTTTCCAGAAGAACTAGGAACCCTGACCAGCGCCATCAACCGGCGGAGCTCAAAACAAAAA |
|  | Reverse | GTGGCAAACCGTATGAGACTCTAGTGAGTCTCAGACTGAACTAGATGAACTCATGCACTGCGCCATCAACCGGCGGAGCTCAAAAATCCAACA |
| UPM51-Selangor | Forward | AAATCATTCCAAGGCCTTCCTCTTTGAGTTCAGAAGAACTAGGAACCCTGACCAGCGCCATCAACCGGCGGAGCTCAAAACAAAAA |
|  | Reverse | ATTTGTGGGAGCTACAACGTAGAGTCTCGGACACAACAGATGCTTCTGACTGCGCGATCGACCGGCGGATCTCTCGATACAACA |
| UPM52-Selangor | Forward | CACCATTTCCCGATGAGGCATCTCTTGAGTTTCAGAAGAACTAGGAACCCTGACCAGCGCCATCAACCGGCGGAGCTCAAAACAAAAA |
|  | Reverse | TCGGAATGCGATGGAGCTCTAGTGAGTCTCGGACAGAACAGAGAACCCTGACTGCGCCATCGACCGGCGGAGCTCTAAAATAAAACAA |
| UPM54-Selangor | Forward | CATGGGCGGCATGAGCATCTCTTGAGTTCAGAAGAACTAGGAACCCTGACCAGCGCCATCAACCGGCGGAGCTCAAAACAAAAA |
|  | Reverse | GACCGTGGGCGATCGAGACTCTAGTACAGTCTCGGACAGAACTAGATGCTCCCATGCACTGCGCCATCAACCGGCGGAGCTCAAAACTAAAACAAG |
| UPM55-Selangor | Forward | GTCAAAAGTCAACGCCTGAGCATCTCTGTAGAGTAGCAGAGAGAACTAGGAACCCTGACCAGCGCCATCAACCGGCGGAGCTCAAAACAAAAA |
|  | Reverse | GGGGCTTAGCATGATCGGGCTTCTACGTACTGTCTCAGGACTCAACTAGGTGGACATCTCCATGCAGCAGTCTGTCCTTTAGTTCGGGACGCGAGCTAAAGCAAATGCGC |
| UPM56-Selangor | Forward | AAAAATCAGCCCCCCTGCGTTGAGCTCTCTTCGAGTTCAGACAGACTAGGAACCCTGACCAGCGCCATCAACCGGCGGAGCTCAAAACAAAAATCCGG |
|  | Reverse | CGGGCCCGTAATGGATACTAGTACAGTCTCGGACTGAACAGTGCTTCTGACTGCGCGATCAACCGGCGGAGCTCAAAACAAAAACA |
| UPM57-Selangor | Forward | CCCTCCCCTTTTAGGCCTCTCTTAGAGTTTTCAGAAGAACTAGGAACCCTGACCAGCGCCATCAACCGGCGGAGCTCAAAACAAAAA |
|  | Reverse | CCCGCCTGGACTCTTACCTAATCGTAGTGTCTCGGACTCACAGATGCTCCCTGCACTGCGCGATCGACCGGCGGAGCTCTAAAATAAAACA |
| UPM58-Selangor | Forward | GACCCTTTCCCGGATGGAGCTTCTCTTGAGTTTCAGAAGACTAGGAACCCTGACCAGCGCCATCAACCGGCGGAGCTCAAAACAAAAA |
|  | Reverse | GTGCATGCGATCGAGCTCTAGTACAGTCTCGGACTGAACTAGATGCTTCATGCACTGCGCCATCAACCGGCGGAGCTCAAAACAAAAACA |
| UPM59-Selangor | Forward | AATTTATCGCCTGGAGTATCTCTTGAGTTTCAGAAGACTAGGAACCCTGACCAGCGCCATCAACCGGCGGAGCTCAAAACAAAAA |
|  | Reverse | ATGGAATGGCGCTGGAGCTCTAGTACAGTCTCGGACAGAACAGATGCTTCTGCACTGCGCGATCGTCGAGCGGATCTCTAAAATAAAACA |
| UPM60-Selangor | Forward | TTGGGCACGTGGCGGTGAGCTCTCTTGAGTTTCAGAAGAACTAGGAACCCTGACCAGCGCCATCAACCGGCGGAGCTCAAAACAAAAAA |
|  | Reverse | TATAGTAGCAGGACGTCAGAGCTCTGTGCAGTCTCGGACAGAACTGGAGAACTCAGCACTGCGCCATCGACCGGCGGTAGCTCTCACTAAAACAT |
| UPM61-Selangor | Forward | ACAGCGAAATAAACGCTGAGCTCTCTTGAGTTTCAGAAGAACTAGGAACCCTGACCAGCGCCATCAACCGGCGGAGCTCAAAACAAAAATTTT |
|  | Reverse | CCCCGCGCGGATAAAGCTCTCGTACAGTCTCGGACACACTAGATGCTCCCTGCACTGCGCGATCGACCAGCGGATCTCTAAGATCCAACAATAGG |
| UPM62-Selangor | Forward | CTTGCGGAATGGTAAGCATCTCTTGAGTTCAGAAGACTAGGAACCCTGACCAGCGCCATCAACCGGCGGAGCTCAAAACAAAAA |
|  | Reverse | TGGCCGGATGAGCTCTAGTGAGTCTCGGACAGACAGTGCTTCTGACAGCGCCATCGACCGGCGGAGCTCTCGATAAAAC |
| UPM63-Selangor | Forward | AAGNAACCATCTCTTGAGTTTCAAGAAAGAACTAGGAACCCTGACCAGCGCCATCAACCGGCGGAGCTCAAAACAAAAAAT |
|  | Reverse | ACGGGCGTGCGAGAGCTCTAGTGAGTCTCGGATGACTGAGATTCTGACTGCGCGATCGTCCGGCCTATTCTCAATAAAACA |
| UPM64-Selangor | Forward | CAGCCAGTTGCCGAATGAGGCATCTCTTGAGTTTCAGAAAGACTAGGAACCCTGACCAGCGCCATCAACCGGCGGAGCTAAAACAAAAA |
|  | Reverse | GAAGTGGCTATCAGCCTCTCGTACAGTCTCGGACAGAACAGTGCTTCTGACTGCGCGATCAACCGGCGGAGCTCAAAACAAAAACAGCGGG |
| UPM65-Selangor | Forward | CCTAAGGGTCGATGAGCATCTCTTGAGTTTCAGAAGACTAGGAACCCTGACCAGCGCCATCAACCGGCGGAGCTCAAAACAAAAAACAC |
|  | Reverse | CAGTTCTCAGAACAGAACTAAATGACATCCCTGCACCAGCGCCATCAACCGGCGGATCTCAAAACTAAAAACAACTCACCTCTCTAGCTTGACGTTT |
| UPM67-Selangor | Forward | CAGTTACCGAGGAGCATCTCTTGAGTTTCAGAAGAACTAGGAACCCTGACCAGCGCCATCAACCGGCGGAGCTCAAAACAAAAA |
|  | Reverse | CATGGCCGATCGGACTCTAGTGAGTCTCGGACAGAACAGATGCTTCTGACTGCGCCATCAACCGGCGGAGCTCTAAAATAAAACA |
| UPM68-Selangor | Forward | CTTTCCCTATCTGTAGGAATGGAGCTCTCTTTGAGTTTCAGAAGAACTAGGAACCCTGACCAGCGCCATCAACCGGCGGAGCTCAAAACAAAAAG |
|  | Reverse | GCGTGGCCGGGATCAGCACTCTAGTGAGTCTCGGACAGAACAGATGCTCCCTGCACTGCGCCATCAACCGGCGGAGCTCAAAACAAAAACA |
| UPM69-Selangor | Forward | ATAACTGCCGGATGGAACCATTCTCTTAGAGTTTCAAGAAAAGACTAGGAACCCTGACCAGCGCCATCAACCGGCGGAGCTCAAAACAAAAA |
|  | Reverse | CGTACATTCGAGCAGACTTCGTAGAGTCTCAGACAGAGTAGTGCTCTCTGCGCTGTCGCGATCGTCCGGCGGAGCTCAAAACAAAAACA |
| UPM70-Selangor | Forward | CGATGAGCTCTCTTGAGTTTCAAGAAAGAACTAGGAACCCTGACCAGCGCCATCAACCGGCGGAGCTCAAAACAAAAATC |
|  | Reverse | CCCGGGCGTAAGCTATCGAGACTCATAGTGCAGTCTCGGACTGAAGTAGGTGGACATCCCATGCGCCAGTCTGTCCATTAGTTCGGACACGCGTACGTCTCATACAGACAAAAACAGCGTCAGGCCCGGATAA |
| UPM82-Selangor | Forward | CCTTGAAGAGGTGCGAAGGAAGCCTCTTCTTGGAGTTTCAGAAAGAACTAGGAACCCTGACCAGCGCCATCAACCGGCGGAGCTCAAAACAAAAA |
|  | Reverse | TTTGAAAAGACTGATCGGCTACTAGTGAGTCTCAGGACACAGTAGATGACTCTCATGCGCTAGTCTGCCATCAACCGGCG  GAGCTCAAAACAAAAACA |
| UPM93-Selangor | Forward | CCAAAAGCGNGAAGCTCTCTTGAGTTTCAAGAAAGAACTAGGAACCCTGACCAGCGCCATCAACCGGCGGAGCTCAAAACA  AAAATTGATTGTNCGGTCT |
|  | Reverse | GCCAGGACGGGAGATCGGGACTCTAGTGCAGTCTCAGAATGAACTAGTGACTCCCATGCACCAGCGCGATCGACCGCACGCTAGTCTCATACAACTAAAACAGTTGCCCTTCTC |
| UPM97-Selangor | Forward | CCAAANGCGAGAAGCATCTCTTGAGTTTCAAGAAAGAACTAGGAACCCTGACCAGCGCCATCAACCGGCGGAGCTCAAAACAAAA |
|  | Reverse | ACGGCCGTACCCGTAAGCTCTAGTGAGTCTCGGACTGAGTAGATGACTCTCCATGCAGCTAGTCTGTCGTATTAGTTCGACACGCTAGTCTCATACAGAATACAAGCAACCAGTAGGGGCCCG |
| UPM100-Selangor | Forward | ACCAAACACCGGCGATGAGCATCTCTTGAGTTTCAGAAGACTAGGAACCCTGACCAGCGCCATCAACCGGCGGAGCTCAAAACAAAAAGC |
|  | Reverse | CCGGCCGCTCAGCGGGTCAAGCTCTAGTGTGTCTCAGGACTGAAGTAGGTGGCTCCCATGCACCAGTCGTCGATTATCCACACGCTAGTCTCATACAAAAAAAACAACTGCTGCGGTGGTA |
| UPM108-Selangor | Forward | CCCACATTCGCGATGAGCTCTCTTGAGTTTCAGAAAGACTAGGAACCCTGACCAGCGCCATCAACCGGCGGAGCTCAAAACAAAAAGTAAAGATG |
|  | Reverse | ACGGATGGCCCGATGAGCTCTAGTGAGTCTCGGACTGACAGATGCTCCCTGACTGCGCCATCGACCGGCGGATCTCTAAACAAAAACAGGT |
| UPM112-Selangor | Forward | CCCTCCTCCGGCGGTATGGAGGCTCTCTTGAGTTTCAGAAAGAACTAGGAACCCTGACCAGCGCCATCAACCGGCGGAGCTCAAAACAAAAACCCTCGGTTCC |
|  | Reverse | ACGGGCAATAGCTGTCGAGCACTAGTGAGTCTCGGACAGAACTGATGCTCCCTGACTAGCGCCATCAACCGGCGGAGCTCAAAACAAAAACAAAAGGCCGAA |
| UPM120-Selangor | Forward | CAAAACTTTAGGCCATGAACGCATCTCTTGAGTTTCAAGAAAGAACTAGGAACCCTGACCAGCGCCATCAACCGGCGGAGCTCAAAACAAAAAGTTCAGT |
|  | Reverse | ACGCCTGGCTGACGAGTTCTAGTGAGTCTCGGACAGACTGTGATCCCTGACAGCGCCATCAACCGGCGGAGCTCTAAACA  AAAACAAG |
| UPM125-Selangor | Forward | CCATGCTGCGGCGGTTGAGCATCTCTTGAGTTTCAAGAAGAACTAGGAACCCTGACCAGCGCCATCAACCGGCGGAGCTC  AAAACAAAAAGAGGC |
|  | Reverse | ACGGGAAGTAGGCTGAAGAGCTCTAGTGAGTCTCGGACTGAACTGATGATCCCTGACCAGCGCCATCAACCGGCGGAGCT  CTAAACAAAAACACCTCCTGT |
| UPM126-Selangor | Forward | CCAAGATCACCCGTTGGAGCATCTCTTGAGTTTCAGAAAGAACTAGGAACCCTGACCAGCGCCATCAACCGGCGGAGCTC  AAAACAAAAAAG |
|  | Reverse | ACGGACTATGCCGATGAGCTCTAGTGAGTCTCAGACTGAACTAATGCTTCTGACCAGCGCCATCAACCGGCGGAGCTCAA  AACAAAAAC |

Supplementary Note: DNA Nucleotide Sequences read for forward and reverse primer which resulting from the sequencing analysis through the sequencing service by First Base database from mosquito pooled samples collected in Kuala Gula Bird Sanctuary, Perak and Kapar Energy Venture, Selangor
