# Supplementary material for: Circulation of West Nile virus in mosquitoes approximate to the migratory bird stopover in West Coast Malaysia
Source: PLoS Negl Trop Dis. 2023 Apr 6;17(4):e0011255. doi: 10.1371/journal.pntd.0011255 (PMC10112790; doi:10.1371/journal.pntd.0011255)
Supplement: S2 Table — (DOCX) [file pntd.0011255.s002.docx]

Supplementary Table S2. List of references strains of WNV used in this study

| No | Accession No | Strain | Country | Year | Host | Lineage |
| --- | --- | --- | --- | --- | --- | --- |
| 1 | KY703856.1 | ArD94343/1992/SN | Senegal | 1992 | *Culex perfuscus* | 1 |
| 2 | MN238670.1 | Lorestna2 | Iran | 2018 | *Culex theileri* | 2 |
| 3 | MN794938.1 | BNI-2615 | Germany | 2019 | *Passer domesticus* | 2 |
| 4 | MN619803.1 | Volgograd829/18 | Russia | 2018 | *Culex pipiens* | 2 |
| 5 | AY765264.1 | Rabensburg isolate 97-103 | Czech Republic | 1997 | *Culex pipiens* | 3 |
| 6 | FJ159129.1 | 101_5-06-Uu | Russia | 2006 | *Uranotaenia unguiculata* | 4a |
| 7 | KJ831223.1 | Uu-LN-AT-2013 | Austria | 2013 | *Uranotaenia unguiculata* | 4c |
| 8 | EU082200.2 | Dak Ar D 5443 | Senegal | 1968 |  | 6 |
| 9 | KY703855.1 | ArD96655/1993/SN | Senegal | 1993 | *Rhipicephalus guilhoni* | 1 |
| 10 | DQ256376.1 | 804994 | India | 1980 | *Homo sapiens* | 5 |
| 11 | MW142226.1 | LE5/2020/Leipzig/Germany | Germany | 2020 | *Homo sapiens* | 2 |
| 12 | MZ964753.1 | CHVir-25910/2021 | Germany | 2021 | *Homo sapiens* | 2 |
| 13 | MW915462.1 | Spain/2017/NG-b | Spain | 2017 | *Accipiter gentilis* | 2 |
| 14 | MW383508.1 | M6848-NA-2020 | Namibia | 2020 | *Culex univittatus* | 2 |
| 15 | MH508038.1 | Buttonwillow_CA_USA/2017-06-28/W494 | USA | 2017 | *Culex tarsalis* | 1 |
| 16 | MN652880.1 | Thessaloniki_MC82m/2018 | Greece | 2018 | *Culex sp* | 2 |
| 17 | AF260969.1 | RO97-50 | Romania | 1997 | *Culex pipiens* | 1 |
| 18 | AF196835.2 | NY99-flamingo382-99 | USA | 1999 | Flamingo | 1 |
| 19 | AF260967.1 | NY99-eqhs | USA | 1999 | horse | 1 |
| 20 | DQ080070.1 | TVP9115 | Mexico | - | Grackel | 1 |
| 21 | AF481864.1 | IS-98 STD | Israel | 1998 | sick stork | 1 |
| 22 | HM152775.1 | WNV_0304h_ISR00 | Israel | 2000 | human | 1 |
| 23 | HM147824.1 | Congo | Congo | 1958 |  | 2 |
| 24 | HM147823.1 | Madagascar | Madagascar | 1988 |  | 2 |
| 25 | HM147822.1 | South Africa | South Africa | 1958 |  | 2 |
| 26 | GQ851603.1 | K6453 | Australia | 1991 | *Culex annulirostris* | 1 |
| 27 | JX041630.1 | LEIV-1640Az | Azerbaijan | 1967 | bird | 1 |
| 28 | EU081844.1 | Egypt 101 | Egypt | - |  | 1 |
| 29 | KC243146.1 | ATH002316 | Kenya | 2010 | *Rhipicephalus pulchellus* | 1 |
| 30 | KF234080.1 | Italy/2009/FIN | Italy | 2009 | human | 1 |
| 31 | JF957186.1 | BSL3-10 | USA | 2010 | - | 1 |
| 32 | JF957184.1 | CO7-09 | USA | 2009 | - | 1 |
| 33 | JF957172.1 | ID28bird-07 | USA | 2007 | bird | 1 |
| 34 | JF957171.1 | ID21bird-07 | USA | 2007 | bird | 1 |
| 35 | GQ903680.1 | Q3574-5 | Cyprus | 1968 | - | 2 |
| 36 | JQ928175.1 | Italy/2011/Piave | Italy | 2011 | human | 1 |
| 37 | JQ928174.1 | Italy/2011/Livenza | Italy | 2011 | human | 1 |
| 38 | JN051153.1 | TM171-03 | Mexico | 2003 | raven | 1 |
| 39 | FJ766332.1 | GE-2o/V | Spain | 2007 | golden eagle | 1 |
| 40 | FJ766331.1 | GE-1b/B | Spain | 2007 | golden eagle | 1 |
| 41 | AF206518.2 | 2741 | USA | 1999 | *Culex pipiens* | 1 |
| 42 | MZ605381.2 | NY99 | USA | 1999 | Flamingo | 1 |
| 43 | MZ605382.4 | Heja | Hungary | 2004 | Goshawk | 2 |
| 44 | MW561633.1 | 769.B/2018/Kavecany/SVK | Slovakia | 2018 | *Strix nebulosa lapponica* | 2 |
| 45 | MT905060.1 | JNESEq804 | Brazil | - | *Equus caballus* | 1 |
| 46 | AF481864 | IS-98 STD | Israel | 1998 | sick stork | 1 |
| 47 | EF429197 | SPU116/89 | South Africa | 1989 | human | 2 |
| 48 | AY277251 | LEIV-Krnd88-190 | Russia | 1998 | *Dermacentor marginatus* | 4a |
| 49 | KC131128.1 | 0121m_ISR09 | Israel | 2009 | mosquito | 2 |
| 50 | KY523178.1 | UG2274/Uganda/2009 | Uganda | 2009 | mosquito pool | 2 |
| 51 | KJ934710.1 | Hyalomma/Romania/2013 | Romania | 2013 | *Hyalomma marginatum marginatum* | 2 |
| 52 | MH986055.1 | 1382/2018/Berlin/Ger | Germany | 2018 | *Turdus merula* | 2 |
| 53 | MK327802 | UPM-Perak33 | Malaysia | 2017 | Little egret | 2 |
| 54 | MK327795 | UPM-Perak6 | Malaysia | 2017 | Black-capped kingfisher | 2 |
| 55 | MK327791 | UPM-Selangor24 | Malaysia | 2017 | Lesser Sand Plover | 2 |
| 56 | MK327787 | UPM-Selangor16 | Malaysia | 2017 | Common Sandpiper | 2 |
| 57 | In this study | UPM59-Perak | Malaysia | 2017 | *Culex tritaeniorhynchus* |  |
| 58 | In this study | UPM5-Selangor | Malaysia | 2019 | *Culex vishnui* |  |
| 59 | In this study | UPM14-Selangor | Malaysia | 2019 | *Culex vishnui* |  |
| 60 | In this study | UPM17-Selangor | Malaysia | 2019 | *Culex vishnui* |  |
| 61 | In this study | UPM23-Selangor | Malaysia | 2019 | *Culex vishnui* |  |
| 62 | In this study | UPM38-Selangor | Malaysia | 2019 | *Culex vishnui* |  |
| 63 | In this study | UPM47-Selangor | Malaysia | 2019 | *Culex vishnui* |  |
| 64 | In this study | UPM49-Selangor | Malaysia | 2019 | *Culex tritaeniorhynchus* |  |
| 65 | In this study | UPM51-Selangor | Malaysia | 2019 | *Culex tritaeniorhynchus* |  |
| 66 | In this study | UPM52-Selangor | Malaysia | 2019 | *Culex tritaeniorhynchus* |  |
| 67 | In this study | UPM54-Selangor | Malaysia | 2019 | *Culex tritaeniorhynchus* |  |
| 68 | In this study | UPM55-Selangor | Malaysia | 2019 | *Culex tritaeniorhynchus* |  |
| 69 | In this study | UPM56-Selangor | Malaysia | 2019 | *Culex tritaeniorhynchus* |  |
| 70 | In this study | UPM57-Selangor | Malaysia | 2019 | *Culex tritaeniorhynchus* |  |
| 71 | In this study | UPM58-Selangor | Malaysia | 2019 | *Culex tritaeniorhynchus* |  |
| 72 | In this study | UPM59-Selangor | Malaysia | 2019 | *Culex tritaeniorhynchus* |  |
| 73 | In this study | UPM60-Selangor | Malaysia | 2019 | *Culex tritaeniorhynchus* |  |
| 74 | In this study | UPM61-Selangor | Malaysia | 2019 | *Culex tritaeniorhynchus* |  |
| 75 | In this study | UPM62-Selangor | Malaysia | 2019 | *Culex tritaeniorhynchus* |  |
| 76 | In this study | UPM63-Selangor | Malaysia | 2019 | *Culex tritaeniorhynchus* |  |
| 77 | In this study | UPM64-Selangor | Malaysia | 2019 | *Culex tritaeniorhynchus* |  |
| 78 | In this study | UPM65-Selangor | Malaysia | 2019 | *Culex tritaeniorhynchus* |  |
| 79 | In this study | UPM67-Selangor | Malaysia | 2019 | *Culex tritaeniorhynchus* |  |
| 80 | In this study | UPM68-Selangor | Malaysia | 2019 | *Culex tritaeniorhynchus* |  |
| 81 | In this study | UPM69-Selangor | Malaysia | 2019 | *Culex tritaeniorhynchus* |  |
| 82 | In this study | UPM70-Selangor | Malaysia | 2019 | *Culex tritaeniorhynchus* |  |
| 83 | In this study | UPM82-Selangor | Malaysia | 2019 | *Culex tritaeniorhynchus* |  |
| 84 | In this study | UPM93-Selangor | Malaysia | 2019 | *Culex tritaeniorhynchus* |  |
| 85 | In this study | UPM97-Selangor | Malaysia | 2019 | *Culex tritaeniorhynchus* |  |
| 86 | In this study | UPM100-Selangor | Malaysia | 2019 | *Culex tritaeniorhynchus* |  |
| 87 | In this study | UPM108Selangor | Malaysia | 2019 | *Culex pseudovishnui* |  |
| 88 | In this study | UPM112-Selangor | Malaysia | 2019 | *Culex pseudovishnui* |  |
| 89 | In this study | UPM120-Selangor | Malaysia | 2019 | *Culex gelidus* |  |
| 90 | In this study | UPM125-Selangor | Malaysia | 2019 | *Armigeres subalbatus* |  |
| 91 | In this study | UPM126-Selangor | Malaysia | 2019 | *Coquillettidia* spp. |  |
