# Supplementary material for: Circulation of West Nile virus in mosquitoes approximate to the migratory bird stopover in West Coast Malaysia
Source: PLoS Negl Trop Dis. 2023 Apr 6;17(4):e0011255. doi: 10.1371/journal.pntd.0011255 (PMC10112790; doi:10.1371/journal.pntd.0011255)
Supplement: Attachment — Submitted filename: PNTD-D-22-00829.review.docx [file pntd.0011255.s003.docx]

***Circulation of West Nile virus in mosquitoes approximate to the migratory bird stopover in West Coast Malaysia***

Jafar Ali Natasha^1^, Abd Rahaman Yasmin^1,2^*, Sharma Reuben Sunil Kumar^3^, Saulol Hamid Nur-Fazila^3^, Md Isa Nur Mahiza^3^, Siti Suri Arshad^3^, Hussni Omar Mohammed^4^, Kiven Kumar^5^, Shih Keng Loong^6^, Mohd Kharip Shah Ahmad Khusaini^7^

^1^Department of Veterinary Laboratory Diagnostics, Faculty of Veterinary Medicine, Universiti Putra Malaysia, 43400 UPM Serdang, Selangor, Malaysia

^2^Laboratory of Vaccines and Biomolecules, Institute of Bioscience, Universiti Putra Malaysia, 43400 UPM Serdang, Selangor, Malaysia

^3^Department of Veterinary Pathology and Microbiology, Faculty of Veterinary Medicine, Universiti Putra Malaysia, 43400 UPM Serdang, Selangor, Malaysia

^4^Department of Population Medicine and Diagnostic Sciences, Cornell University, Ithaca, New York, USA

^5^Department of Pathology, Faculty of Medicine and Health Sciences, Universiti Putra Malaysia, 43400 UPM, Serdang, Selangor, Malaysia

^6^Tropical Infectious Diseases Research and Education Centre, University of Malaya, 50603, Kuala Lumpur, Malaysia

^7^Department of Conservation of Biodiversity of Wildlife and National Park Malaysia, Jalan Cheras, 56100, Kuala Lumpur, Malaysia

*Corresponding author:

E-mail: noryasmin@upm.edu.my (ARY)

**Abstract**

Being a tropical country with a conducive environment for mosquitoes, mosquito-borne illnesses such as dengue, chikungunya, lymphatic filariasis, malaria, and Japanese encephalitis are prevalent in Malaysia. Recent studies reported asymptomatic infection of West Nile virus (WNV) in animals and humans, but none of the studies were conducted in mosquitoes, except for one report made half a century ago. Considering the scarcity of information, our study sampled mosquitoes near migratory water and resident wild birds at the Kuala Gula Bird Sanctuary and Kapar Energy Venture. Our previous publication reported that those birds were positive for WNV antibody and RNA. Using a nested RT-PCR analysis, WNV RNA was detected in 35 (12.8%) also see Akhtaof 285 mosquito pools comprised of 2,635 mosquitoes, most of which were *Culex* species. Sanger sequencing and phylogenetic analysis revealed that the squences grouped in lineage 2 and shared 90.12%–97.01% similarity with sequences found locally as well as those from Africa, Germany, Romania, Italy, and Israel. Evidence of WNV in the mosquitoes further substantiates the need for continued surveillance of WNV in Malaysia.

**Synopsis**

West Nile virus (WNV) infection is considered to be one of the neglected diseases in Malaysia as most vector-borne febrile illnesses in humans are predominantly diagnosed as dengue-like illnesses. Nevertheless, WNV might be one of the culprits causing the mosquito-borne disease in Malaysia based on the data from previous studies. Our group previously identified the presence of WNV RNA and antibodies in the migratory and resident birds, macaques, bats and swine. However, there is no current data that could prove the presence of WNV in the vector, responsible for transmission of WNV to the wide range of hosts. Using molecular analysis, WNV RNA was detected from six mosquito species, namely *Culex tritaeniorhynchus*, *Culex vishnui*, *Culex pseudovishnui*, *Culex gelidus*, *Armigeres subalbatus*, and *Coquillettidia* spp. Additionally, the sequence of WNV RNA was similar with the WNV lineage isolated from wild birds at the same location. These findings highlight the risk of WNV infection among people and animals in Malaysia.

**Introduction**

WNV is an emerging zoonotic pathogen that is transmitted by primarily *Culex* mosquitoes causing significant outbreaks in birds, humans, and other mammals such as horses globally [3,4]. WNV is an envelope RNA virus with genome size of approximately 11kb, is grouped in the *Flaviviridae* family [5].

The abundance of mosquito vectors combined with avian migration has exacerbated the dispersal and dynamics of WNV [6]. Some migratory birds are competent to maintain viremias even after a long flying distances and then transmit virus to mosquitoes.Ornithophilic mosquitoes such as *Culex* species aquire the virus from these birds during the blood mealand then transmit the virus to native naïve birds and other susceptible hosts [7]. Animals and humans have an equal risk of acquiring mosquito-borne diseases, which will increase the rate of emerging and re-emerging diseases globally [8].

Malaysia is located in the tropics, an ideal environment for mosquito diversity and abundance [9]. The first report of WNV isolation was in 1960 from *Culex pseudovishnui* collected in Sarawak, Borneo [10]. In addition, WNV RNA and/or antibodies were discovered in multiple species, including companion birds, water birds, migratory birds, swine, bats, and macaques as well as in humans [11–13]. However, the current status of WNV in mosquitoes, in Peninsular Malaysia is lacking. Identifying possible vector species and sites that have a greater risk of WNV transmission are essential to plan for the prevention of WNV outbreaks in future. The current study was carried out at the Kuala Gula Bird Sanctuary and Kapar Energy Venture, areas where WNV RNA and antibodies previously were detected in migratory and resident waterbirds [11]. Mosquitoes reported here were collected simultaneously with that previous study; however, additional time was needed for the taxonomic identification of the specimens before virus detection, so the data was not presented together. The discovery of WNV infection in mosquito populations in these locations is a crucial first step to understand the WNV vector status of mosquito species in the region.

**Methods**

**Sampling consent and approval**

A study approval from Institutional Animal Care and Use Committee (IACUC) was received from Universiti Putra Malaysia under reference number UPM/IACUC/AUP NO: R043/17. The approval from the Department of Wildlife and National Parks (DWNP), Peninsular Malaysia, was granted, with the research permit number JPHL&TN(IP):100-6/1/14 to collect mosquitoes from the wild bird landing areas.

**Study design and sampling location**

A cross-sectional study design was conducted to identify the species diversity of the mosquito population near wild bird habitats at the Kuala Gula Bird Sanctuary in the Perak state (100.49° E latitude and 4.94° N longitude) and Kapar Energy Venture in the Selangor state (101.32° E latitude and 3.12° N longitude). These sites were regarded as high risk for WNV due to the availability of migratory birds. The study location map was extracted and prepared using Google Earth Pro version 7.3.3. (Fig 1).

**Fig 1. Sampling sites for mosquitoes.** The sampling sites are marked with a yellow pin. Both sites face the Straits of Malacca. Temperatures were recorded for the nights of mosquito collection.

Geographically, both sites are surrounded by mangrove trees and adjacent to the sea. Kuala Gula Bird Sanctuary and Kapar Energy Venture, which face the Straits of Malacca, are a frequentstopover site for migratory birds [14]. Ringing (leg banding) of migrants is conducted by staff of the Department of Wildlife and National Parks (DWNP). Kuala Gula Bird Sanctuary is also covered by paddy fields where residential areas and water bird landing could be found [11,15]. Kapar Energy Venture contains an ash pond considered safe for water birds [14]. Both are rich in mudflats and water bodies, a favored environment for the mosquitoes.

**Mosquito collection**

Mosquitoes were collected during two consecutive nights on October 2017 at Kuala Gula Bird Sanctuary and on __ September 2019 at Kapar Energy Venture bird habitats. Temperaturewas obtained from the official website of the Malaysian Meteorological Department (<https://www.met.gov.my/>) [16] (Fig 1). Two Miniature CDC Light Trap Model 512 with a UV light (John W. Hock Company, USA) suspended at 1.5 m height and baited with dry ice (Linde, Malaysia) were

were operated overnight from around 1800h until about 0700h.. The collecting bag containing mosquitoes were placed in an icebox and covered with a damp cloth as suggested by Coleman et al. [18] to keep the collected mosquitoes alive. Upon reaching the laboratory, the collecting bags were stored at −80°C (Sanyo, Japan) at the Laboratory of Virology, Faculty of Veterinary Medicine, Universiti Putra Malaysia, for further analysis.

**Mosquito identification and analysis**

**Mosquito identification**

Mosquitoes were sorted on a chilled pan based on taxonomic keys and morphological characteristics described by Becker et al. [2], Jeffery et al. [19], and Jourdain et al. [20 and then pooled by genus, sex, date, and collection site in a range of one to ten (1–10) mosquitoes Pools were stored at –80°C (Sanyo, Japan) in the Laboratory of Virology, Faculty of Veterinary Medicine, Universiti Putra Malaysia

**Mosquito relative abundance**

The total number of mosquitoes from each habitat was tabulated with Microsoft Excel. Mosquito species prevalence was calculated as:

$$Relative abundance=\frac{Number of each species mosquito}{Total number of mosquitoes} \times100$$

Abundance of more than 5% wasconsidered as a dominant species, less than 5% as subdominant species, and less than 1% considered as satellite species [24].

**Species Diversity Analysis**

The species diversity was determined using the Palaeontological Statistics (PAST) software. Four diversity indices were calculated, i.e., the Margalef and Simpson indices, to analyse the heterogeneity and species richness of the mosquitoes for each selected habitat [25]. The value of the index reflects the hierarchy of diversity, ranging from low, medium, and high [26]. According to Ceretti-Junior et al. [27], greater species diversity in a habitat is indicated by a higher diversity index, i.e., greater than 1.

**Molecular analysis**

**Mosquito homogenization**

The homogenization procedures were followedPatsoula et al. [21]. The process was conducted in a Biosafety Cabinet Class 2 (ESCO, Singapore). About 500 µL of phosphate buffer saline (PBS) was added to every pooled mosquito sample. The homogeniser was sterilised with 500 µL of 70% alcohol and PBS for 3 to 5 min before and after sample homogenisation. The pooled mosquito samples were then homogenised under 805 × *g* using a battery-operated pellet pestle cordless motor (Sigma, USA) with an autoclavable plastic pestle (Biobasic, Canada) in a container filled with crushed ice until the mosquito was fully macerated.

**RNA extraction and quantification**

Total RNA extraction of the homogenised mosquitoes was performed using Trizol (Bioline, UK), according to the manufacturer’s protocol. The concentration and purity of extracted RNA were quantified using the Biophotometer (Eppendorf, Hamburg, Germany) at 260 nm wavelength. RNA purity recorded between 1.8 and 2.1 is considered pure RNA.

**Nested Reverse Transcriptase – Polymerase Chain Reaction (Nested RT-PCR)**

A nested reverse transcriptase-polymerase chain reaction (Nested RT-PCR) assay was conducted on a MasterCycler Gradient thermal cycler (Eppendorf, Germany) to screen for WNV from the extracted RNA. The first and second round of Nested RT-PCR reaction consisted of a 25 µL total standard reaction, with 3 µL and 2 µL of template, respectively. The synthetic plasmid gene of WNV capsid and pre-membrane was used as the positive control in this study [13]. Two sets of primers targeting a conserved region of WNV, namely capsid and pre-membrane gene, were used (Table 1).

**Table 1. Sequences of Primers Used in This Study**

|  | Primer | Sequence (5’ – 3’) | Amplicon Size (bp) | Reference |
| --- | --- | --- | --- | --- |
| First Round | Forward | CCAATACGTTTCGTGTTGG | 437 | Najwa et al. (2020) |
|  | Reverse | GGAAATGACCCTGAAGACAT |  |  |
| Second Round^a^ | Forward | GCTGGATCGATGGAGAGGTG | 114 | - |
|  | Reverse | CGGCGGAGCTCAAAACAAAA |  |  |

^a^The second-round primer was designed with reference strain: KX547617.

In this study, the second-round primer set was designed through the NCBI primer designing tool (https://www.ncbi.nlm.nih.gov/tools/primer-blast/). The application was acquired from the NCBI network resources centre with the accession number KX547617. A set of primers amplifying the similar targeted gene with the positive control (between WNV capsid and pre-membrane gene) was designed using the Primer Blast tool (<http://www.ncbi.nlm.nih.gov/tools/primer-blast/>), with the expected base pair size of 114 bp.

The extracted RNA sample was subjected to One-Step Reverse Transcriptase Polymerase Chain Reaction (RT-PCR; Bioline, UK) as the first round of PCR. The volume of each PCR reagent for one reaction is shown in Table 2. The total reaction of the amplification mixture for the first round of PCR was vortexed and placed in the thermal cycler (Eppendorf, Germany). The first-round protocol was performed under the cycling condition as follows: reverse transcription at 95°C for 20 min, polymerase activation at 95°C for 1 min, 40 cycles of denaturation at 95°C for 10 s, annealing at 52°C for 10 s, and extension at 72°C for 30 s, which proceeded to the final extension at 72°C for 5 min and on hold for infinity at 10°C.

The PCR product was used as the template for the second round of PCR. MyTaq RedMix kit (Bioline, UK) was used in the second round of PCR. The total volume of the master mix for the second round is summarised in Table 2. The total reaction of the second round of PCR is as follows: initial denaturation at 95°C for 1 min, 30 cycles of denaturation at 95°C for 15 s, annealing at 52°C for 15 s, extension at 72°C for 10 s, and put on hold at 10°C for infinity.

**Table 2. Total Volume of PCR Master Mix for The First and Second Round of PCR**

| First Round of PCR |  | Second Round of PCR |  |
| --- | --- | --- | --- |
| Reagent | **Volume (µL)** | **Reagent** | **Volume (µL)** |
| Deionized distilled water | 12.0 | **MyTaq Red Mix** | 12.5 |
| MyTaq One Step Mix | 8.0 | **Deionized distilled water** | 9.5 |
| Forward Primer^a^ | 0.5 | **Forward Primer^b^** | 0.5 |
| Reverse Primer^a^ | 0.5 | **Reverse Primer^b^** | 0.5 |
| Reverse Transcriptase | 0.5 | **-** | - |
| RiboSafe Rnase Inhibitor | 0.5 | **-** | - |
| Template | 3.0 | **Template** | 2.0 |
| TOTAL | 25.0 |  | 25.0 |

The total volume of both PCR was optimized in prior for the determination of annealing temperature

^a^10µM of primer were applied in first round of PCR (437bp)

^b^20µM of primer were applied in first round of PCR (114bp)

**Gel electrophoresis**

The nested PCR products were visualised through gel electrophoresis using 1.5% agarose gel and Red Safe (iNTRON Biotechnology, Korea) as a pre-cast stain. The agarose gel was prepared by mixing 0.5 g of agarose powder (HR agarose, USA) and 100 mL of Tris-Acetate-EDTA (TAE) buffer (Sigma Aldrich, USA). About 2 µL of 100 bp Hyperladder (Bioline, UK), which serves as the molecular weight marker, were loaded into the second well of the solidified agarose gel. The gel was electrophoresed at 90 V with 400 mA (Bio-Rad power supply, USA) for 30 min. The amplicon was visualised with a UV transilluminator (Syngene, UK), and the gel image was captured using software (Syngene, UK).

**DNA sequencing**

**Partial DNA sequencing**

The positive amplicon from the nested RT-PCR assay was subjected to partial DNA sequencing. Nucleospin Gel and PCR Clean-up kit (Macherey-Nagel, Germany) was used to purify positive PCR product, followed by DNA sequencing analysis using ABI PRISM 3730*xl* Genetic Analyser (Applied Biosystems, USA). Samples with positive bands acquired from the nested PCR analysis were sent for DNA sequencing (Apical Scientific, Malaysia).

**Basic Local Alignment Search Tool (BLAST)**

The nucleotide sequence (S1 Table) of the positive band samples was run through the Basic Local Alignment Search Tool (BLAST) database of the NCBI nucleotide sequence. The BLAST was applied to determine the percentage similarity of WNV with nucleotide sequences from other regions.

**Phylogenetic analysis**

The sequences were aligned using the Multiple Sequence Alignment (MAFFT) software version 7, yielding 64 WNV sequences (S2 Table), including 10 sequences from this study. The phylogenetic tree was built using the maximum likelihood method with the Jukes-Cantor model in the Molecular Evolutionary Genetics Analysis (MEGA) 7. A bootstrapped confidence interval with 1000 replicates was set. The Newick file was created, and the tree was viewed and edited using the FigTree.v1.4.4.

**Result**

**Mosquito identification**

**Mosquito distribution**

Overall, 2,635 mosquitoes were collected from Kuala Gula Bird Sanctuary, Perak (1,417 mosquitoes, 53.78%) and Kapar Energy Venture, Selangor (1,218 mosquitoes, 46.22%). The number of pools per species is shown in Table 3.

The total mosquitoes from both bird habitats were comprised of 2621 (99.47%) female mosquitoes and 14 (0.99%) male mosquitoes (Table 3A total of 11 genera of mosquitoes were identified based on morphological keys (Table 3), with2,611 out of 2,635 mosquitoes (98.71%) in the genus *Culex*. Only one mosquito each was identified in *Aedemomyia*, *Anopheles*, *Coquilettidia*, *Heizmannnia*, *Mansonia*, *Topomyia*, and *Uranotaenia* .

**Subgenera and species Identification**

Two subgenera were identified for the subgenus identification of the *Culex* genus mosquitoes, i.e., *Culex* subgenus *Culex* and *Culex* subgenus *Lophoceraomyia* found in both bird habitats.In total, 19 species were discovered, with seven belonging to the *Culex* genus, including *Culex tritaeniorhynchus*, *Culex vishnui*, *Culex pseudovishnui*, *Culex quinquefasciatus*, *Culex gelidus*, and *Culex* spp. (Fig 2). The appearance of black erect scales on the vertex and scutum ranges from white to dark brown to black scales, representing the species of the *Culex* genus. *Cx. tritaeniorhynchus* was identified by the dark to black scales, with the ventral part of the proboscis outstretching proximally, differentiating it from other *Culex* species.

**Fig 2. Venn diagram of the identified mosquito species in selected bird habitats.** Each circle presents the mosquito species found in Kuala Gula Bird Sanctuary (green) and Kapar Energy Venture, Selangor (pink). The overlapped circle presents the mosquito species found in both habitats (brown).

The morphology classification for the *Cx. vishnui* species is the dark brown vertex scale on top of the head, with parts of the scutum covered with dark brown scales. In contrast, the taxonomic key of *Cx. pseudovishnui* is the light to brown scales on the scutum and pale scales on the vertex.

The apparent feature of *Cx. gelidus* is the scutum, located on the dorsal part of the thorax that is covered with white scales up to the prescutellar area at the ventral of the scutum. In comparison, *Cx. quinquefasciatus* has striped colour on the abdomen. The basal area is paler than the apical area, accompanied by the absence of a pale and dark pattern in the mesokatepisternum and mesepimeron site on the lateral part of the thorax.

**Relative abundance**

Three *Culex* species were dominant in CO2 baited light traps in Kuala Gula Bird Sanctuary in Perak and Kapar Energy Venture in Selangor. The species are *Cx*. *tritaeniorhynchus*, *Cx. vishnui*, and *Cx. pseudovishnui* (Table 3). Meanwhile, *Cx. gelidus* (2.54%) is denoted as the sub-dominant species in Kuala Gula Bird Sanctuary, Perak, but classified as satellite species in Kapar Energy Venture due to the number of less than 1% (3/1218). The remaining mosquito species from *Culex* and other genera are also identified as satellite species.

*Cx. tritaeniorhynchus* exhibits the highest number of bird habitats in this study, i.e., Kuala Gula Bird Sanctuary (996 out of 1417, 70.29%) and Kapar Energy Venture (550 out of 1218, 45.16 %). Similarly, *Cx. vishnui*, found in both studied bird habitats, is the second most abundant mosquito after *Cx. tritaeniorhynchus*, with 480 mosquitoes (39.41%) at Kapar Energy Venture, Selangor, two times more than in Kuala Gula Bird Sanctuary, with 279 mosquitoes (19.70%). *Culex pseudovishnui* were identified about 84 out of 1417 mosquitoes (6.21%), in Kuala Gula Bird Sanctuary, Perak, while 154 out of 1218 mosquitoes were from Kapar Energy Venture, Selangor. Whereas *Culex gelidus* showed a remarkable number of only female mosquitoes in Kuala Gula Bird Sanctuary (36/1417) at 2.54% compared to Kapar Energy Venture, Selangor, with 3/1218 at 0.25%.

**Diversity indices**

Based on the calculated diversity indices using the PAST software, mosquito species diversity in Kapar Energy Venture are greater than in Kuala Gula Bird Sanctuary. Four indices were applied in this study. The obtained values for all indices are shown in Table 4. The Margalef index in Kapar Energy Venture is higher than Kuala Gula Bird Sanctuary, indicating a greater species richness of mosquito species. The diversity t-test showed no significant difference in the Simpson index between Kuala Gula Bird Sanctuary and Kapar Energy Venture (t = −5.99, p-value = 2.25). Similarly, no significant difference is noted for the Simpson Index in both bird habitats (t = 10.56, p-value = 1.94). The Simpson Index of Kapar Energy Venture is higher than Kuala Gula, indicating that Kapar Energy Venture has a higher diversity of mosquitoes due to more species at the site.

**Table 4. Diversity Indices between Kuala Gula Bird Sanctuary and Kapar Energy Venture bird habitats**

| Habitat | Kuala Gula Bird Sanctuary | Kapar Energy Venture |
| --- | --- | --- |
| Margalef Index, M | 1.654 | 1.689 |
| Dominance, D | 0.537 | 0.375 |
| Simpson Index, (1-D) | 0.463 | 0.625 |
| Shannon Index, H | 0.913 | 1.134 |

These indices to compare the species richness of selected bird habitats in Kuala Gula Bird Sanctuary and Kapar Energy Venture.

**Detection of WNV RNA**

The total collected mosquito from the bird habitats was divided into 285 mosquito pools (Table 3). WNV RNA was detected in 35 out of 285 (12.28%) mosquito pools (Fig 3). The positive WNV pool of mosquitoes belongs to six different species, i.e., *Cx. tritaeniorhynchus* (24 pools), *Cx. vishnui* (6 pools), *Cx. pseudovishnui* (2 pools), *Cx. gelidus* (1 pool), *Armigeres subalbatus* (1 pool), and *Coquillettidia* spp. (Table 3). All the positive mosquito pools comprise female mosquitoes, with 1 out of 24 positive mosquito pools *of Cx. tritaeniorhynchus* was found in Kuala Gula Bird Sanctuary, Perak, while the remaining species of positive mosquito pools were found in Kapar Energy Venture, Selangor.

**Figure 3. Bands of positive mosquito samples from the gel electrophoresis procedure**. The expected target size is 114 bp, targeting capsid and pre-membrane genes of the WNV. The gel was prepared with 1.5% agarose gel electrophoresis (w/v). Key: L: 100 bp size ladder; −ve ctrl: negative control (deionised distilled water); +ve ctrl: positive control (WNV plasmid); S1–S5: pooled samples of female *Cx. vishnui* mosquitoes.

**DNA Sequencing**

**Partial DNA Sequencing and BLAST**

Partial sequences (forward and reverse) of positive amplicons from this study were combined using the BioEdit software (version 7.2.5.0) after generating the reverse complement strands. Positive WNV RNA pools of mosquitoes were confirmed with the partial DNA sequencing analysis using BLAST tools. The isolates from this study have a high similarity with Malaysian strains from Perak and Selangor, ranging between 97.01% and 92.01%, and South African strain with 92.59% homology. Most of the strains are approximately 91.36% identical to Germany (MH986055), Romania (KJ934710), Uganda (KY523178), and Israel (KC131128) strains. Meanwhile, the strain from this study is 90.12% similar to the Italian strain (MN939557 and MN939564).

**Phylogenetic analysis**

A phylogenetic tree constructed using the maximum likelihood method with the Jukes-Cantor model showed evolutionary relationships of taxa of WNV positive isolates from this study. The positive isolates are grouped with WNV strains from the WNV lineage 2 (Fig 4). All positive amplicons from this study share the same branch, which diverged from other lineage 2 WNV sequences.

**Fig 4. Phylogenetic analysis of WNV detected in the mosquito pools in Malaysia with other global WNV.** The analysis involved 64 nucleotide sequences, including 10 sequences from this study (highlighted in red). The tree branches are colored according to the proposed WNV lineages; light green: Lineage 1; orange: Lineage 2; other lineages (3, 4a, and 5): purple. The tree was constructed using the maximum likelihood method with the Jukes-Cantor model with 1000 bootstrap replicate. Branches are scaled bootstraps.

**Table 3. Mosquito Distribution According to Location, Genus, Species and Pool Number**

| Habitat | Genus | Species | Sex | | RA (%) | Total Mosquito by Species | Number of mosquitoes per pool | Number of pools | Positive WNV RNA pools | Accession Number |
| --- | --- | --- | --- | --- | --- | --- | --- | --- | --- | --- |
|  |  |  | **M** | **F** |  |  |  |  |  |  |
| Kuala Gula Bird Sanctuary, Perak | *Aedeomyia (Aedeomyia)* | *catastica* |  | 1 | 0.07 | 1 | 1 | 1 | - |  |
|  | *Aedes (Aedes)* | *albopictus* |  | 6 | 0.42 | 6 | 6 | 1 | - |  |
|  | *Armigeres (Armigeres)* | *kesseli* |  | 1 | 0.07 | 1 | 1 | 1 | - |  |
|  | *Armigeres (Armigeres)* | *maximus* |  | 1 | 0.07 | 1 | 1 | 1 | - |  |
|  | *Culex (Culex)* | *gelidus* |  | 36 | 2.54 | 36 | 10 | 4 | - |  |
|  |  | *pseudovishnui* | 4 | 84 | 6.21 | 88 | 10 | 10 | - |  |
|  |  | *tritaeniorhynchus* | 2 | 994 | 70.29 | 996 | 10 | 101 | 1 | ON809701 |
|  |  | *quinquefasciatus* | 2 | 2 | 0.28 | 4 | 2 | 2 | - |  |
|  |  | *vishnui* | 5 | 274 | 19.70 | 279 | 10 | 29 | - |  |
|  | *Culex (Lophoceraomyia*) | *spp.* | 1 | 1 | 0.14 | 2 | 1 | 2 | - |  |
|  | *Mansonia (Mansonioides)* | *annulifera* |  | 1 | 0.07 | 1 | 1 | 1 | - |  |
|  | *Uranotaenia* | *spp.* |  | 1 | 0.07 | 1 | 1 | 1 | - |  |
|  | *Verralina (Verralina)* | *butleri* |  | 1 | 0.07 | 1 | 1 | 1 | - |  |
| Kapar Energy Venture, Selangor | *Aedes (Aedes)* | *albopictus* |  | 2 | 0.16 | 2 | 2 | 1 | - |  |
|  | *Anopheles (Cellia)* | *spp.* |  | 1 | 0.08 | 1 | 1 | 1 | - |  |
|  | *Armigeres (Armigeres)* | *subalbatus* |  | 2 | 0.16 | 2 | 2 | 1 | 1 | TBR |
|  | *Coquillettidia (Coquillettidia)* | spp. |  | 1 | 0.08 | 1 | 1 | 1 | 1 | ON815484 |
|  | *Culex (Culex)* | *gelidus* |  | 3 | 0.25 | 3 | 3 | 1 | 1 | ON815483 |
|  |  | *pseudovishnui* |  | 154 | 12.64 | 154 | 10 | 16 | 2 | ON815482 |
|  |  | *tritaeniorhynchus* |  | 550 | 45.16 | 550 | 10 | 55 | 23 | TBR |
|  |  | *vishnui* |  | 480 | 39.41 | 480 | 10 | 48 | 6 | OM945733; ON809702 |
|  | *Culex (Lophoceraomyia* | *spp.* |  | 1 | 0.08 | 1 | 1 | 1 | - |  |
|  |  | *spp.* |  | 18 | 1.48 | 18 | 10 | 2 | - |  |
|  | *Heizmannia (Heizmannia)* | *spp.* |  | 1 | 0.08 | 1 | 1 | 1 | - |  |
|  | *Topomyia* | *spp.* |  | 1 | 0.08 | 1 | 1 | 1 | - |  |
|  | *Verralina (Verralina)* | *butleri* |  | 4 | 0.33 | 4 | 4 | 1 | - |  |
| Total | | | **14** | **2621** |  | **2635** |  | **285** | **35** |  |

M is indicated as male mosquitoes while F is indicated as female mosquitoes. RA is indicated as relative abundance. TBR is indicated as to be retrieved. Maximum number of pools per species is 10 while minimum is 1.

**Discussion**

In the west coast of Peninsular Malaysia, Kuala Gula Bird Sanctuary and Kapar Energy Venture are the typical areas where migratory birds stopover from northern and southern flyways in Malaysia [14]. This area is also a cohabitation locality of vast bird species, from domestic water birds to wild birds to roost. As these species continue to contribute important ecological values in the ecosystem, yet they are renowned as the principal reservoir of fatal infection of WNV and JEV. On the other hand, mosquitoes play an important role in perpetuating and maintaining the viruses by acting as the transmission vector to another susceptible host [28]. Najwa et al. [11], who conducted the sampling of wild birds’ sera and oropharyngeal swabs in the same location as this study, revealed that WNV antibody was detected in 29/155 birds (18.71%), while WNV RNA was detected from 16/105 birds (15.2%). These findings are alarming because knowing the role of mosquitoes in the WNV transmission, the absence of data on mosquito status in WNV infection here, renders the crucial necessity and importance of this study. Therefore, this study was aiming to report the current data and findings on the diversity of the mosquitoes collected in the area and their WNV status.

Criteria such as sex, age, and genera of mosquitoes and the duration of sampling were emphasised in this study. Meanwhile, the time frame from twilight until the next dawn is the roosting hour for birds and the period where ornithophilic mosquitoes, especially *Culex* spp. or night biter, are more active [29,30]. Female adult mosquitoes are preferred in this study because only adult female mosquitoes that feed on host blood are responsible for virus transmission both vertically and horizontally [31-32]. In addition, female mosquitoes usually prefer the smell of the host as the major attractant during blood feeding. On the other hand, male mosquito prefers visual attractant during feeding on the nectar [33]. To produce the smell attractant in the CDC light trap, dry ice that produces CO_2_ was used. CO_2_ will mimic the exhaled gas of the host, which is one of the female mosquito’s odour attractants [34]. Therefore, this study utilised CDC light trap baited with CO_2_ to capture predominantly female adult mosquitoes.

The selection of mosquito trapping method is important in contributing to the abundance of mosquito species and, importantly, fits the purpose of the study [35]. For instance, CDC light trap is one of the methods used effectively to capture adult mosquitoes [35]. This has been proven by a study by Sriwichai et al. [17], who discovered that the CDC light trap is the best trap that gave significant numbers of adult mosquitoes during the collection. [35]. Several studies have highlighted that CDC light trap is also an effective tool to capture the Culex spp. [36]. Since *Culex* genera have been recognised as the principal vector for WNV transmission [37-39], this study chose a method of trapping that captures more *Culex* species.

Data from entomological study based on the mosquito population in regard to their relative abundance and diversity will aid in the designing effective mosquito surveillance and vector control programme [40]. In this study, *Culex* mosquitoes are the dominant species found near bird habitats. Chaturanga et al. [41] discovered similar finding, which is anticipated due to the ornithophilic properties of *Culex* mosquitoes that prefer bird’s blood during feeding [29]. The *Culex* genus was captured with other genera because the selected habitat contain high population of mosquitoes as the environment is very conducive for the mosquitoes to survive effectively [36,42].

Nested RT-PCR was performed to determine the WNV RNA in the homogenised mosquitoes by targeting the conserved regions of WNV between the capsid and pre-membrane protein regions. This type of PCR is highly sensitive as opposed to conventional PCR, which has been used to detect the presence of WNV from lineage 1 and 2 [43-45]. The nested RT- PCR assay revealed that 35 out of the 285 pools of mosquitoes were positive for WNV RNA (12.28%), which is dominantly from *Culex* mosquitoes. Other studies have also reported the presence of WNV in *Cx. tritaeniorhynchus* in Assam, India [44]. In other regions in Xinjiang, China and Greece, WNV was also detected from the *Culex* genera, i.e., *Cx*. *pipiens* 10.17% (12/118) [46,47]. Additionally, Jiang et al. [48] revealed that *Cx.* *tritaeniorhynchus* was among the competent vector of WNV through an experimental study in China.

The sequencing analysis revealed that the mosquito isolates in this study showed approximately 97.01% similarity with WNV isolated from wild birds at the same study site and interestingly similar to South African and American crow, all originating from lineage 2. Findings from these studies indicated that WNV lineage 2 might be circulating in Malaysia, indicating the significant role played by the migratory birds during flyaway to other continents. Studies have demonstrated that wild bird territory might pose a high-risk area for WNV and JEV transmission [5,49].

Despite having a humid climate due to their location in rural and coastal areas, these areas might serve as high-risk mosquito production sites surrounded by paddy fields and mangroves. Malaysia experience two monsoon wind seasons, i.e., the Southwest Monsoon (summer season) from April to September and the Northeast Monsoon (rainy season) from October to March [50]. The sampling of mosquitoes in this study was conducted in October during the rainy season. Usually, during this season, extended surface water is formed in the water body that supports mosquito breeding [51,52]. With increased humidity, mosquitoes survived longer and dispersed further, contributing to increased chances of WNV transmission among the mosquitoes and the wild birds.

The sampling of mosquitoes from this study was conducted simultaneously with wild birds in Kuala Gula. Wild birds from this area showed positive WNV RNA and antibodies [11]. Besides, the JEV antibody was also detected in wild birds in this area [53]. Other than the orofecal direct transmission of WNV among birds, the ornithophilic characteristic of the mosquitoes increases the chance of wild bird exposure to various *Culex* spp. mosquitoes as the mosquitoes were found to be attracted to the high body temperature of the birds, ranging from 41°C to 42°C [54]. Besides, temperatures between 27°C and 28°C enhanced WNV transmission [55].

The wild bird is the important reservoir host for WNV, which consists of a significant amount of WNV in the blood to continue the replication cycle of WNV [56]. Besides, birds and mosquitoes generate vector-host interaction, which helps increase the evolution of WNV [57]. Therefore, mosquitoes are the most potential vector of WNV, and the detection of WNV antigen among mosquitoes provides a meaningful outcome for future research about WNV.

**Conclusion**

In summary, findings from the current study indicated that WNV is evident in most *Culex* species, as demonstrated by the detection of WNV RNA from the nested PCR analysis. This study further signifies the role played by the *Culex* species as the main vector of WNV. Remarkably, the origin of WNV isolated from mosquitoes and bird habitats originated from the same lineage, i.e., WNV lineage 2, suggesting that this lineage circulates in Malaysia. A further entomological investigation is required to describe the feeding and breeding preferences, virus surveillance, and mutations. Imperative monitoring programmes targeting to eliminate the transmission of WNV through vector control programmes, public awareness, and biosurveillance of pathogens in wild birds are being implemented via one health initiative among different authorities. This study is also in line with Malaysia Strategy for Emerging Diseases and Public Health Emergencies (MYSED) II (2017–2021) under the Ministry of Health, which prioritised enhancing regional capability to rapidly and accurately survey, detect, diagnose, and report outbreaks of pathogens and diseases of security concern.

**Acknowledgements**

We would like to thank the Department of Wildlife and National Parks (DWNP), Peninsular Malaysia, for the sampling approval. The greatest appreciation is attributed to the late Mr John Jeffery, the writer of entomology books, for his guidance and help in the mosquito works.

**References**

1. Mosquito Info. [cited 20 June 2022]. In The American Mosquito Control Association [Internet]. Sacramento. Available from: <https://www.mosquito.org/page/mosquitoinfo>
2. Becker N, Zgomba M, Boase C, Madon M, Dahl C, Kaiser A. Mosquito and their control. 2nd ed. New York: Springer Heidelberg Dordrecht; 2010.
3. Petersen LR, Brault AC, Nasci RS. West Nile virus: review of the literature. JAMA. 2013; 310: 308-315.
4. Asnis DS, Conetta R, Waldman G, Teixeira AA. The West Nile virus encephalitis outbreak in the United States (1999-2000) from Flushing, New York, to beyond its border. Ann N Y Acad Sci. 2001; 95: 161-171.
5. Zhang X, Zhang Y, Jia R, Wang M, Yin Z, Cheng A. Structure and function of capsid protein in flavivirus infection and its applications in the development of vaccines and therapeutics. Vet Res. 2021; 52: 98.
6. Maquart M, Boyer S, Rakotoharinome VM, Ravaomanana J, Tantely ML, Heraud JM, et al. High prevalence of West Nile virus in domestic birds and detection in 2 new mosquito species in Madagascar. PLoS One. 2016; 11: e0147589.
7. Farajollahi A, Fonseca DM, Kramer LD, Kilpatrick AM. “Bird biting” mosquitoes and human disease: a review of the role of *Culex pipiens* complex mosquitoes in epidemiology. Infect Genet Evol. 2011; 11: 1577-1585.
8. Wu X, Lu Y, Zhou S, Chen L, Xu B. Impact of climate change on human infectious diseases: empirical evidence and human adaptation. Environ Int. 2016; 86: 14-23.
9. Zainon N, Mohd Rahim FA, Roslan D, Abd Samat AH. Prevention of *Aedes* breeding habitats for urban high-rise building in Malaysia. Plans Malays. 2016; 14: 115-128.
10. Bowen ETW, Simpson DIH, Platt GS, Way HJ, Smith CG, Ching CY, et al. Arbovirus infections in Sarawak: the isolation of Kunjin virus from mosquitoes of the *Culex pseudovishnui* group. Ann Trop Med Parasitol. 1970; 64: 263-268.
11. Ain-Najwa MY, Yasmin AR, Omar AR, Arshad SS, Abu J, Mohammed HO, et al. Evidence of West Nile virus infection in migratory and resident wild birds in west coast of peninsular Malaysia. One Health. 2020; 10: 100134.
12. Ain-Najwa MY, Yasmin AR, Arshad SS, Omar AR, Abu J, Kumar K, et al. Exposure to zoonotic West Nile virus in long-tailed macaques and bats in peninsular Malaysia. Animals. 2020; 10: 2367.
13. Mohammed MN, Yasmin AR, Ramanoon SZ, Arshad SS, Bande F, Mohammed HO. Serological evidence of West Nile viral infection in archived swine serum samples from peninsular Malaysia. J Vet Sci. 2021; 22: e29.
14. Bakewell D. Kapar Power Station Waterbird Report 2008. Kuala Lumpur, Malaysia: MNS-Bird Conservation Council. MNS-BCC Waterbirds Group Report No.1. 2009 [Cited 2022 June 20]. Available from: <http://mnswetland.weebly.com/uploads/3/1/8/0/31801977/kapar_power_station_waterbird_report_2008_final.pdf>.
15. Rahman F, Ismail A, Shohaimi S, Omar H, Hussin MZ. Foraging activity of the re-introduced milky storks (*Mycteria cinerea*) in Kuala Gula Bird Sanctuary, Perak, Malaysia. Pertanika J Trop Agric Sci. 2020; 43: 217-229.
16. General Climate in Malaysia. [cited 20 June 2022]. In: Official Website of Malaysian Meterological Malaysia [Internet]. Selangor 2021. Available from: <https://www.met.gov.my/>
17. Sriwichai P, Karl S, Samung Y, Sumruayphol S, Kiattibutr K, Payakkapol A, et al. Evaluation of CDC light traps for mosquito surveillance in a malaria endemic area on the Thai Myanmar border. Parasit vector. 2015; 8: 636.
18. Coleman WB, Tsongalis GJ. Molecular Pathology: The molecular basis of human diseases. In: Williams ES, Silverman LM, editors. Molecular diagnosis of human disease. 2018. pp. 691-707.
19. Jeffery J, Rohela M, Muslimin M, Abdul Aziz SMN, Jamaiah I, Kumar S, et al. Illustrated keys: some mosquitoes of peninsula Malaysia, Malaysia. 1st ed. Malaysia: University of Malaya Press; 2012.
20. Jourdain F, Picard M, Sulesco T, Haddad N, Harrat Z, Sawalha SS, et al. Identification of mosquitoes (Diptera: Culicidae): an external quality assessment of medical entomology laboratories in the MediLabSecure Network. Parasit vector. 2018; 11: 553.
21. Patsoula E, Beleri S, Tegos N, Mkrtsian R, Vakali A, Pervanidou D. Entomological data and detection of West Nile virus in mosquitoes in Greece (2014–2016), before disease re-emergence in 2017. Vector Borne Zoonotic Dis. 2020; 20: 60-70.
22. Ramírez AL, Hall-Mendelin S, Doggett SL, Hewitson GR, McMahon JL, Ritchie SA, et al. Mosquito excreta: a sample type with many potential applications for the investigation of Ross River virus and West Nile virus ecology. PLoS Negl Trop Dis. 2018; 12: e0006771.
23. Condotta SA, Hunter FF, Bidochka MJ. West Nile virus infection rates in pooled and individual mosquito samples. Vector Borne Zoonotic Dis. 2004; 4: 198-203.
24. Trojan P. The analysis of the fauna’s structure. Memorabilia Zool. 1992; 47: 1-120.
25. Khoobdel M, Keshavarzi D, Mossa-Kazemi SH, Sobati H. Species diversity of mosquitoes of the Genus *Culex* (Diptera, Culicidae) in the coastal areas of the Persian Gulf. AIMS Public Health. 2019; 6: 99-106.
26. Varghese AO, Menon ARR. Floristic composition, dynamics and diversity of Myristica swamp forests of Southern-Western Ghats, Kerala. Indian For. 1999; 125: 775-783.
27. Ceretti-Junior W, Oliveira-Christe R, Wilk-da-Silva R, Mucci LF, de Castro Duarte AMR, Fernandes A, et al. Diversity analysis and an updated list of mosquitoes (Diptera: Culicidae) found in Cantareira State Park, São Paulo, Brazil. Acta Trop. 2020; 212: 105669.
28. Anderson, SL, Richards SL, Smartt CTA. A simple method for determining arbovirus transmission in mosquitoes. J Am Mosq Control Assoc. 2010; 26: 108-111.
29. González MA, Prosser SW, Hernández-Triana LM, Alarcón-Elbal PM, Goiri F, López S, et al. Avian feeding preferences of *Culex pipiens* and *Culiseta* spp. along an urban-to-wild gradient in northern Spain. Front Ecol Evol. 2020; 8: 568835.
30. Kauffman E, Payne A, Franke MA, Schmid MA, Harris E, Kramer LD. Rearing of *Culex* spp. and *Aedes* spp. mosquitoes. Bio Protoc. 2017; 7: e2542.
31. Kweka EJ, Baraka V, Mathias L, Mwang’onde B, Baraka G, Lyaruu L, et al. Ecology of *Aedes* mosquitoes, the major vectors of arboviruses in human population. In: Abelardo Falcón-Lezama J., Betancourt-Cravioto M., Tapia-Conyer R, editors. Dengue fever – a resilient threat in the face of innovation. London: IntechOpen; 2018. pp. 39-55.
32. Silver JB. Mosquito ecology: field sampling methods. 3rd ed. New York: Springer Heideberg Inchelhit; 2018.
33. Wolbachia-*Aedes* Mosquito Suppression Strategy. [cited 20 June 2022]. In: National Environment Agency [Internet]. Singapore. Available from: <https://www.nea.gov.sg/corporate-functions/resources/research/wolbachia-aedes-mosquito-suppression-strategy/male-mosquitoes-do-not-bite>
34. Dormont L, Mulatier M, Carrasco D, Cohuet, A. Mosquito attractants. J Chem Ecol. 2021; 47,: 351-393.
35. Boyer S, Tantely ML, Randriamaherijaona S, Andrianaivolambo L, Cardinale E. Mosquitoes sampling strategy for studying West Nile virus vectors in Madagascar: abundance, distribution and methods of catching in highrisk areas. Arch Inst Pasteur Madagascar. 2014; 71: 1-8.
36. Hutchinson RA, West PA, Lindsay SW. Suitability of two carbon dioxide-baited traps for mosquito surveillance in the United Kingdom. Bull Entomol Res. 2007; 97: 591-597.
37. Balenghien T, Vazeille M, Grandadam M, Schaffner F, Zeller H, Reiter P, et al. Vector competence of some French *Culex* and *Aedes* mosquitoes for West Nile virus. Vector Borne Zoonotic Dis. 2008; 8: 589-595.
38. Engler O, Savini G, Papa A, Figuerola J, Groschup MH, Kampen H, et al. European surveillance for West Nile virus in mosquito populations. Int J Environ Res Public Health. 2013; 10: 4869-4895.
39. Vogels CB, Göertz GP, Pijlman GP, Koenraadt CJ. Vector competence of European mosquitoes for West Nile virus. Emerg Microbes Infect. 2017; 6: e96.
40. Schwab SR, Stone CM, Fonseca DM, Fefferman NH. The importance of being urgent: the impact of surveillance target and scale on mosquito-borne disease control. Epidemics. 2018; 23: 55-63.
41. Chathuranga WGD, Karunaratne SHPP, Fernando BR, De Silva WPP. Diversity, distribution, abundance, and feeding pattern of tropical ornithophilic mosquitoes. J Vector Ecol. 2018; 43: 158-167.
42. Dale PER, Knight JM. Wetlands and mosquitoes: a review. Wetl Ecol Manag. 2008; 16: 255-276.
43. Ergunay K, Gunay F, Erisoz Kasapj O, Oter K, Gargari S, Karaoglu T, et al. Serological, molecular and entomological surveillance demonstrates widespread circulation of West Nile virus in Turkey. PLoS Negl Trop. Dis. 2014; 8: e3028.
44. Khan SA, Chowdhury P, Choudhury P, Dutta P. Detection of West Nile virus in six mosquito species in synchrony with seroconversion among sentinel chickens in India. Parasit Vector. 2017; 10: 13.
45. Shi PY, Kauffman EB, Ren P, Felton A, Tai JH, Dupuis AP, et al. High-throughput detection of West Nile virus RNA. J Clin Microbiol. 2001; 39: 1264-1271.
46. Lu Z, Fu SH, Cao L, Tang CJ, Zhang S, Li ZX, et al. Human infection with West Nile virus, Xinjiang, China, 2011. Emerg Infect Dis. 2014; 20: 1421-1423.
47. Mavridis K, Fotakis EA, Kioulos I, Mpellou S, Konstantas S, Varela E, et al. Detection of West Nile Virus–Lineage 2 in *Culex pipiens* mosquitoes, associated with disease outbreak in Greece, 2017. Acta Trop. 2018; 182: 64-68.
48. Jiang SF, Zhang YM, Guo XX, Dong YD, Xing D, Xue RD. Experimental studies on comparison of the potential vector competence of four species of Culex mosquitoes in China to transmit West Nile virus. J Med Entomol. 2010; 47: 788-790.
49. Pérez-Ramírez E, Llorente F, Jiménez-Clavero MÁ. Experimental infections of wild birds with West Nile virus. Viruses. 2014; 6: 752-781.
50. Jamaluddin AF, Khazainani S, Mohd Hairy I, Mohd Azizi MT, Norhadizah MK, Chung SJ, et al. Iklim Perak. Malaysia: Malaysian Meteorological Department; 2019.
51. Basari N, Aiman Syazwan H, Mohd Zairi Z, Nur Aida H. Larval distributions and breeding habitats of *Aedes aegypti* and *Ae. albopictus* in Kuala Terengganu. Trop Biomed. 2016; 33: 420-427.
52. Seidahmed OM, Eltahir EA. A sequence of flushing and drying of breeding habitats of *Aedes aegypti* (L.) prior to the low dengue season in Singapore. PLoS Negl Trop Dis. 2016; 10: e0004842.
53. Kumar K, Arshad SS, Selvarajah GT, Abu J, Toung OP, Abba Y, et al. Prevalence and risk factors of Japanese encephalitis virus (JEV) in livestock and companion animal in high-risk areas in Malaysia. Trop Anim Health Prod. 2018; 50: 741-752.
54. Kumar K, Arshad SS, Selvarajah GT, Abu J, Toung O.P, Abba Y, et al. Japanese encephalitis in Malaysia: An overview and timeline. Acta Trop. 2018; 185: 219-229.
55. Benbetka S, Hachid A, Benallal KE, Benbetka C, Khaldi A, Bitam I, et al. First field evidence infection of *Culex perexiguus* by West Nile virus in Sahara Oasis of Algeria. J Vector Borne Dis. 2018; 55: 305-309.
56. Dietrich EA, Langevin SA, Huang CYH, Maharaj PD, Delorey MJ, Bowen RA, et al. West Nile Virus temperature sensitivity and avian virulence are modulated by NS1-2B polymorphisms. PLoS Negl Trop Dis. 2016; 10: e0004938.
57. Rizzoli A, Tagliapietra V, Cagnacci F, Marini G, Arnoldi D, Rosso F, et al. Parasites and wildlife in a changing world: The vector-host-pathogen interaction as a learning case. Int J Parasitol Parasites Wildl. 2019; 9: 394-401.

**Supporting Information Captions**

**S1 Fig. CDC Light Trap setting baited with dry ice.**

**S2 Fig. Lateral view of mosquito for sex determination of mosquito.**

**S3 Fig. Microscopic image on the anterior view of the genus *Aedeomyia*, *Aedes*, *Anopheles*, and *Armigeres*,**

**S4 Fig. Microscopic image on the lateral view of the genus *Coquillettidia*, *Culex*; *Heizmannia*, and *Mansonia***

**S5 Fig. Microscopic image on the anterior view of the genus *Topomyia*, *Uranotaenia*, and *Verralina***

**S1 Table. Partial DNA Sequence of Positive Isolates of WNV RNA from Mosquitoes**

**S2 Table. List of Reference strain of WNV used in this study.**
