# Supplementary material for: Circulation of West Nile virus in mosquitoes approximate to the migratory bird stopover in West Coast Malaysia
Source: PLoS Negl Trop Dis. 2023 Apr 6;17(4):e0011255. doi: 10.1371/journal.pntd.0011255 (PMC10112790; doi:10.1371/journal.pntd.0011255)
Supplement: Attachment — Submitted filename: Rebuttal Letter and List of Response to Review Comments .docx [file pntd.0011255.s004.docx]

02^nd^ October 2022

Editor-in-Chief,

PLOS Neglected Tropical Disease,

Shaden Kamhawi,

Vector Molecular Biology,

National Institutes of Health,

Rockville, MD, USA

Dear Editor,

REVISED MANUSCRIPT FOR PUBLICATION

Enclosed herewith is a revised manuscript entitled “Circulation of West Nile Virus in Mosquitoes Approximate to the Migratory Bird Stopover in West Coast Malaysia” for publication in PLOS Neglected Tropical Disease.

We thank reviewers for suggestions and comments. We the feel that the reviewer's comments helped clarify and improve the manuscript. The response to the comments is attached. With the submission of this manuscript, I would like to undertake that the above-mentioned manuscript has not been published elsewhere, accepted for publication elsewhere, or under editorial review for publication elsewhere.

I hope the manuscript is in satisfactory condition and has the merit for publication in PLOS Neglected Tropical Disease.

Thank you in advance.

With best regards,

------------------------------------------------

Dr. Nor Yasmin Abd. Rahaman

Corresponding author

E-mail: [noryasmin@upm.edu.my](mailto:noryasmin@upm.edu.my)

**Response to Reviewer 1**

Dear Reviewers,

The authors have revised the manuscript accordingly and feel that the reviewer's comments helped clarify and improve the manuscript. Please find our responses.

| **No.** | | **Reviewer’s comments** | **Response** | **Line** | **Page** |
| --- | --- | --- | --- | --- | --- |
| 1. | | **Editorial and Data Presentation Comments**  The attached manuscript was edited extensively through L214 [see attached file]. In general, the paper was written in a very 'loose fashion' and contained excessive details that should be carefully eliminated where possible. Much of the semantics will require careful scrutiny during revision so terms such as vector, host, reservoir, etc are specific. The paper will need complete revision to reduce length to a short communication. This can be done, in part, by limiting the extensive presentation of the entomological data | The authors would like to express great appreciation for the revision and editing of the manuscript. The comments were taken into account seriously and amended accordingly.  In regard to short communication format, PLOS NTDs does not currently publish short communications articles, therefore the authors hope that the current revised manuscript that has been improved in methodology, results and discussion has been suggested by the reviewers, could be submitted as a research article. | - | - |
| 2. | | Their data indicated a very high minimum infection rate in *Cx. tritaeniorhynchus* from one sampling site on one occasion several years ago. It would be useful to determine if this was a consistent event and why transmission was so high at this one site. | As this study is a collaborative work with government agencies, a series of surveillance of the mosquitoes will be one of the follow up plans for this work. | - | - |
| 3. | | The paper was too long for the amount of field work done and should be reduced to a short communication focusing on the high infection rates and how this related to data from their previous avian papers. | The authors would like to express great appreciation for the revision and editing of the manuscript. The comments were taken into account seriously and amended accordingly. In regard to short communication format, PLOS NTDs does not currently publish short communications articles, therefore the authors hope that the current manuscript could be submitted as a research article. | - | - |
| 4. | | Which avian species had the highest infection rates and were the mosquitoes collected near their nesting/perching sites? *Cx. tritaeniorhynchus* typically feed more frequently on large mammals than birds [see Reisen and Boreham 1979 AJTMH 28: 408; and other papers], so these results were indeed interesting from that perspective. Perhaps more details about host availability near trap deployment sites would be revealing? | The authors would like to thank the reviewer for the comment. The information about host availability near the trap deployment sites has been added as suggested by the reviewer. The authors agreed that feeding patterns for *Cx. Tritaeniorhynchus* typically feed more to large mammals as studied by Reisen and Boreham 1979 AJTMH 28: 408; and Fall et al [2011]. However, the same study Fall et al [2011], demonstrated that apparent *Cx. tritaeniorhynchus* densities near to pigeon habitat started to increase dramatically following arrival of migratory birds in the region and showed a declining pattern over the time. <https://doi.org/10.1186/1756-3305-4-99>. Together with the recognized role as a competent vector of WNV and timing of this study that coincides with migratory birds' arrival, explains why WNV has relatively high prevalence in this mosquito species. The information about the avian species that had the highest infection rates has been included as well. | Methodology. Line  143  Discussion. Line  351-360 | 6  18 |
| 5. | | Limited sampling effort. If I read this correctly, mosquitoes were collected at two sites on separate occasions in different years and months using two traps set for two consecutive nights; total effort = 8 trap nights. This is very limited sampling effort and certainly does not lend itself to the extensive analysis of diversity and abundance. These parts of the paper should be deleted. | The authors would like to thank the reviewer for the comment. Considering the point raised, we have deleted the parts on diversity and abundance. | - | - |
| 6. | | Mosquito identification. Although this could be do to their presentation, it seemed that mosquitoes were frozen, sorted to sex/genus on a chill table, pooled, refrozen, ground and RNA extracted and tested. Later we learn the specimens were sorted by taxa -- some by species -- before pooling. It was not clear when this was done? | The authors would like to thank the reviewer for the comment. It is true that the collected mosquitoes were frozen twice, the first was upon collection in the field. But later it was refrozen after the identification and pooling procedure. The explanation has been added accordingly. | Methodology. Line  160 | 7 |
| 7. | | The authors included descriptions of the different Culex species that was not necessary as this has been published by various systematists [although the authors did not cite these publications -- e.g., Sirivanakarn 1976.Medical entomology studies III.A revision of the subgenus Culex in the oriental region (Diptera:Culicidae) Contr. Am. Entomol. Inst. 12: 1- 271]. | The authors would like to thank the reviewer for the comment. The suggested reference by the reviewer was included in the ‘Illustrated Key Mosquito in Peninsular Malaysia’ book [17] (Jeffery et al., 2012) that was applied for mosquito identification in this study. | - | - |
| 8. | | As all specimens were tested for virus, there were no voucher specimens saved for species confirmation and confirmation was not done by molecular methods -- this could be problematic as the 3 abundant *Culex* species are difficult sort, expecially when rubbed in traps or wet on a chill table. | The authors would like to thank the reviewer for the comment. The species collected in this study was verified in person by the experienced entomologist, who is also the writer of the ‘Illustrated Key Mosquito in Peninsular Malaysia’ book [17] (Jefferry et al., 2012). | Methodology. Line  162 | 7 |
| 9. | | Virus detection. The authors used a nested RT-PCR to detect virus. Too much detail was given on the methods used which were adopted from previous studies. | The authors would like to thank the reviewer for the comment. The first set of primers was adopted from a previous study. The Round 2 PCR however was modified and designed specifically for more sensitive detection of the virus. | Methodology. Line  195 | 8 |
| 10. | | Yes, However, much of this 2 was excessive for the data collected and therefore most of the Figures were not necessary | Table 2 was deleted as suggested by the reviewer. | - | - |
| 11. | Vague – this implies there have been no attempts to isolate/detect viruses in mosquitoes for the past 50 years? What about endemic JEV? | | The authors would like to thank the reviewer for the comment. This statement is actually referring to attempt to detect WNV in Malaysia. | Abstract. Line  35 | 2 |
| 12. | Were these pools cultured and the viruses now ‘in hand’ or were they just sequenced? | | The authors would like to thank the reviewer for the comment. The positive pools were utilised for both purposes. The attempts to isolate the virus were unsuccessful in cell culture, probably due to longer storage before the isolation work was started. The positive pools RNA is currently being studied for FGS. | - | - |
| 13. | Infection is not necessarily an illness? | | The authors would like to thank the reviewer for the comment. The ‘infection’ word was deleted | - | - |
| 14. | No need to remind the PNTD readers of this. | | The authors would like to thank the reviewer for the comment. The suggestion is accepted and was deleted | - | - |
| 15. | Not all! Especially some of the anseriforms. | | The authors would like to thank the reviewer for the comment. The information has been edited to be more specific by stating the exact group of susceptible birds (which is passerine species) as suggested by the reviewer. | Introduction, Line  73 | 3 |
| 16. | Data? References? This supposition has been hard to prove. | | The authors would like to thank the reviewer for the comment. The reference was added as suggested by the reviewer. | Introduction, Line  75 | 3 |
| 17. | Confusing…. Disease is the response of the host to infection and each species has a different response? | | The author apologizes for the confusion. This sentence implying the zoonotic properties of the virus. | Introduction, Line  77 | 3 |
| 18. | Refrence? Seems to deal with Aedes control? | | The authors would like to thank the reviewer for the comment. A new reference has been added with justification. | Introduction, Line  82 | 4 |
| 19. | Correct – these are political states? | | The authors would like to thank the reviewer for the comment. These states are a federation state | Methodology. Line  117 | 5 |
| 20. | Unclear? | | The authors would like to thank the reviewer for the comment. The sentences are referring to geographical information of sampling collection and have been rephrased. | Methodology. Line  130 | 6 |
| 21. | Do people live here? Other larval habitats or hosts such pigs? | | The authors would like to thank the reviewer for the comment. The explanation about the location has been added as suggested by the reviewer. | Methodology. Line  125 | 5 |
| 22. | Tidal? Brackish water? | | The authors would like to thank the reviewer for the comment The sentence was amended accordingly | Methodology. Line  126 | 5 |
| 23. | Give dates | | The authors would like to thank the reviewer for the comment The date of sampling has been added. | Methodology. Line  134 | 6 |
| 24. | How many traps operated per night? | | The authors would like to thank the reviewer for the comment. Two traps were operated per night | Methodology. Line  134 | 6 |
| 25. | Rest of paragraph is standard methods and not needed. | | The authors would like to thank the reviewer for the comment The sentence has been deleted | - | - |
| 26. | This is really simple diversity and not really relative abundance. | | The authors would like to thank the reviewer for the comment. Considering the point raised, we have deleted the parts on diversity and abundance. | - | - |
| 27. | I feel this is over analysing very little collection effort. Traps are biased for those respecies responding to the trap. You only sampled for 4 trap-nights at two sites 2 years apart! | | The authors would like to thank the reviewer for the comment. Considering the point raised, we have deleted the parts on diversity and abundance. | - | - |
| 28. | Your methods are too detailed especially is you followed somebody’s protocol??? | | The authors would like to thank the reviewer for the comment. The author apologizes for the confusion. The second-round primer was designed by the author except for reagent buffer used and the preferences on mosquito pooled number. | Methodology. Line  164 | 7 |
| 29. | This paper studies aby in swine? | | The authors would like to thank the reviewer for the comment The references was deleted and replaced with the correct number | Methodology. Line  186 | 8 |
| 30. | Not in references? Use number format | | The authors would like to thank the reviewer for the comment. The references were amended and replaced with the correct format. | Methodology. Line  188 | 8 |
| 31. | Lineage? | | The authors would like to thank the reviewer for the comment. The information has been added. | Methodology. Line  190 | 8 |
| 32. | Not needed | | The authors would like to thank the reviewer for the comment. The table has been deleted | - | - |
| 33. | Not needed. Why not examine the Terminalia? Not all species have very different antennae/palps. | | The authors would like to thank the reviewer for the comment. In this study, the examination of antenna and size of palps were used for sex determination. Since the method of trapping used was mainly to capture female mosquitoes, we did not include examination of the Terminalia. Nevertheless, we found occasionally the gravid status of the abdominal part of the female mosquito during the identification under the microscope, but since the criteria was not included in the examination, the recording was not jot down. | Methodology. Line  158 | 7 |
| 34. | Not necessary to give species descriptions in this paper. Would have been useful to use molecular confirmation of your | | The authors would like to thank the reviewer for the comment. The mosquito homogenate unfortunately has been entirely used up for the virus work. We believe using molecular methods is one of the best options for the species determination. Having said that, the morphological determination used in this study has been tutored and verified by the experienced entomologist, who is also the writer of the ‘Illustrated Key Mosquito in Peninsular Malaysia’ book [17] (Jefferry et al., 2012). | Methodology. Line  162 | 7 |
| 35. | Based on the methods these were identified to Culex, pooled and ground for viral extraction. When were the species identified and how? Not clear how this was done if the pools were already mixed????? | | The authors would like to thank the reviewer for the comment. The explanation has been added accordingly in the manuscript as suggested by the reviewer. After collection, the identification of the mosquitoes were conducted, then pooled before RNA was extracted. | Methodology. Line  166 | 7 |
| 36. | How were these identified? Adult females can be very difficult, especially is wet on a chill table? | | The authors would like to thank the reviewer for the comment. The explanation has been added accordingly in the manuscript as suggested by the reviewer. The examined mosquito was placed on the clean dry petri dish, while the ice was on another petri dish and placed below the examined petri dish mosquito. | Methodology. Line  160 | 7 |
| 37. | Not needed | | The figure was deleted as suggested by the reviewer. | - | - |
| 38.. | Including vishnui? | | Based on the the morphology characterisation is specific to *Culex tritaeniorhynchus;* ‘Illustrated Key Mosquito in Peninsular Malaysia’ book [17] (Jefferry et al., 2012). | Methodology. Line  158  Result  Line  233 | 7  10 |
| 39. | Delete – samples were taken in different months in different years and therefore cannot be directly compared, especially when some species were collected so infrequently. | | The authors would like to thank the reviewer for the comment. Considering the point raised, we have deleted the parts on diversity and abundance. | - | - |
| 40. | delete | | The authors would like to thank the reviewer for the suggestion. Considering the point raised, we have deleted the parts on diversity and abundance. | - | - |
| 41. | delete | | The authors would like to thank the reviewer for the suggestion. Considering the point raised, we have deleted the parts on diversity and abundance. | - | - |
| 42. | So it appears from Fig 4 than only 10 of the 35 pools were sequenced and confirmed? | | The authors would like to thank the reviewer for the comment. All 35 pools were sequenced and has been included in the phylogenetic analysis. All the isolates from this study were grouped together in one cluster, and closely related with lineage 2. | Result.  Line  281 | 12 |
| 43. | Isn’t this just similarity Lineage 2? Which strains were Kunjin showing affinity to Australia? | | The authors would like to thank the reviewer for the comment. The Kunjin virus was based on published papers [ Bowen ETW, Simpson DIH, Platt GS, Way HJ, Smith CG, Ching CY, et al. Arbovirus infections in Sarawak: the isolation of Kunjin virus from mosquitoes of the Culex pseudovishnui group. Ann Trop Med Parasitol. 1970; 64: 263-268] | Result.  Line  268 | 12 |
| 44. | Hard to find additional Malaysia sequences? | | The authors would like to thank the reviewer for the comment. The additional Malaysia sequences was included in the phylogenetic analysis and the labelling has been improved and stated as wild birds from Malaysia. | Result.  Line  282 | 12 |
| 45. | Reference? | | The reference was added accordingly | Result.  Line  307 | 16 |
| 46. | Unclear? | | The sentence has been rephrased to be a more clear description. | Result.  Line  317 | 16 |
| 47. | Thereby compromising diversity analysis! | | The authors would like to thank the reviewer for the comments. Considering the point raised, we have deleted the parts on diversity and abundance. | - | - |
| 48. | Minimum infection rate was 13.3/1,000 tested – very high. | | The authors agreed that MIR is high in this study. Based on the findings from the Nature scientific report by Chakraburti and Smith, 2019; doi: <https://doi.org/10.1038/s41598-019-55632-7>, low sampling trap density increases the potential for error in MIR estimation, and that it does so synergistically with true MIR values. | Methodology. Line  218  Result.  Line  289  Discussion.  Line  362-369 | 9  13  18 |
| 49. | Also see Akhter et al. 1982. TRSTMH 76:449. Cx. trit. Was thye most susceptible of the species tested. | | The authors would like to thank the reviewer for the comment. The reference has been added accordingly | Discussion. Line  352 | 18 |
| 50. | Were these included in Fig. 4? | | The authors would like to thank the reviewer for the comment. The labelling has been improved and stated as wild birds from Malaysia (presented in the blue box) in Figure 3 | Result.  Line  281  Discussion. Line  371-379 | 12  18 |
| 51. | Vewry general? Which species coming from which continents? | | The authors would like to thank the reviewer for the comment. The sentences have been amended. | Discussion. Line  390 | 19 |
| 52. | Unclear if the venture site had paddy field? Most of the Culex do not do well in brackish/tidal water and require fresh water. | | The authors would like to thank the reviewer for the comment. This study [<https://citeseerx.ist.psu.edu/viewdoc/download?doi=10.1.1.1027.5183&rep=rep1&type=pdf>] presents almost similar pattern of *Culex* sp as our study. | Discussion. Line  382 | 19 |
| 53. | And September – see methods | | The authors would like to thank the reviewer for the comment. The sentence has been amended. | Discussion. Line  383 | 19 |
| 54. | Data? References? | | The authors would like to thank the reviewer for the comment. The information has been added accordingly | Discussion. Line  384 | 19 |
| 55. | Why were the mosquitoes negative for this virus? Or was the nested RT-PCR too specific to detect it? | | The authors would like to thank the reviewer for the comment. The statement was referring to JEV. The study failed to detect the presence of virus RNA at that particular time in the different animal species. The type of RT-PCR used was conventional single round RT-PCR, not nested RT-PCR. The sample used for RT-PCR was serum which often has low level and transient viraemia, which might explain the reason for negative detection of Kumar et al. study. | - | - |
| 56. | There are other factors to consider before this is confirmed. | | The authors would like to thank the reviewer for the comment. The sentences has been amended | Conclusion. Line  406 | 20 |

**Response to Reviewer 2**

Dear Reviewer,

The authors have revised the manuscript accordingly and feel that the reviewer's comments helped clarify and improve the manuscript. Please find our responses.

| **No.** | **Reviewer’s comments** | **Response** | **Line** | **Page** |
| --- | --- | --- | --- | --- |
| 1. | One overall criticism is that very little data are being presented in this study. Several sections, and especially the methods and discussion sections, are excessively large. Normally I would expect this kind of study to be presented as a short communication, but I’m not sure if PLOS NTD has such an article type. Some of the figures and tables are also excessive | The authors would like to express great appreciation for the revision and editing of the manuscript. The comments were taken into account seriously and amended accordingly. In regard to short communication format, PLOS NTDs does not currently publish short communications articles, therefore the authors hope that the current revised manuscript that has been improved in methodology, results and discussion as been suggested by the reviewers, could be submitted as a research article. | - | - |
| 2. | The infection rate is not presented (but should be) but based on the percent of the positive pools, it appears to be very high. | The minimum infection rate information was added in the manuscript as suggested by the reviewer. Based on the findings from the Nature scientific report by Chakraburti and Smith, 2019; doi: <https://doi.org/10.1038/s41598-019-55632-7>, low sampling trap density increases the potential for error in MIR estimation, and that it does so synergistically with true MIR values. | Methodology. Line  218  Result.  Line  289  Discussion. Line  363 | 9  13  18 |
| 3. | The mention that these mosquitoes were collected in October only comes up in the discussion and needs to be presented earlier. The bird and mosquito infection with WNV in this region in October is remarkably high (almost higher than what would be biologically possible for WNV). I would assume the enzootic cycle has seasonality associated with it so did the authors just happen to sample during the peak amplification period in a WNV hotspot? This topic of seasonality deserves more attention. | The authors would like to thank the reviewer for the comment and the details about the collection month and weather have been added as suggested by the reviewer. | Methodology. Line  134-137  Discussion. Line  381 | 6  19 |
| 4. | The authors appear to be fixated on natural wetland areas that have wild migratory birds. However, WNV circulates in urban residential areas in much of the world, facilitated by the presence of Culex mosquitoes and avian amplification hosts (e.g. house sparrows, etc.). Is there a chance the authors are overlooking WNV transmission in other landscapes, including urban, in Malaysia? | In Europe, *Culex sp.* has almost the same preference over urban versus rural area [<https://doi.org/10.1093%2Fjisesa%2Fiex086>].  While in the US, *Culex sp.* mostly prefer urban as opposed to rural areas. [<https://doi.org/10.1186/s13071-021-05051-3> ].  As in Malaysia, *Culex sp*. was predominantly abundant in rural and sub-rural area. In fact, outbreak of Japanese Encephalitis (*Culex sp.* is the vector) in Malaysia with fatal human case was often reported in rural area [<https://www.thestar.com.my/news/nation/2021/11/29/two-fatal-cases-of-je-infection-in-kota-setar-district-says-kedah-health-director>].  In addition to that, the rural area which is the migratory bird stopover was preferred in this study primarily because the information on whether *Culex* mosquitoes in this habitat is carrying WNV is lacking despite the WNV RNA and antibodies found in the bird reservoir in the same area. Since this is a pioneer study, subsequent study in other landscapes is absolutely a crucial need. | Methodology. Line  125 | 5 |
| 5. | The manuscript contains poor English and has inappropriate scientific language. I started making suggestions for specific to the abstract and then stopped providing suggestions for the main body as they were extensive. Given the combination of poor English with poor use of scientific language, extensive edits by a relevant expert are necessary. | The authors would like to thank the reviewer for the editing and revision, which indeed is a great help in improving this manuscript.  The manuscript has been proofread as suggested by the reviewer. | - | - |
| ~~3~~6. | These study sites could be explained where they are. Malaysia? Coastal | The authors would like to thank the reviewer for the comment and the details about the location has been added. | Abstract.  Line  37  Methodology. Line  125 | 2  6 |
| 7. | Macaque should be plural like the rest. | The authors would like to thank the reviewer for the comment. The word has been amended accordingly | Synopsis. Line  61 | 3 |
| 8. | Replace ‘prove’ with ‘confirm’. | The authors would like to thank the reviewer for the comment. The word has been amended accordingly | Synopsis. Line  62 | 3 |
| 9. | Change to: “WNV to a wide range of hosts” | The authors would like to thank the reviewer for the comment. The sentence has been amended accordingly. | Synopsis. Line  63 | 3 |
| 10. | ‘WNA’ should be ‘WNV’. | The authors would like to thank the reviewer for the comment. The word has been amended accordingly | Synopsis. Line  63 | 3 |
| 11. | Could be revised to: “Thus, these findings confirm the presence of an enzootic cycle of WNV in Malaysia and risk for spill-over to humans and animals.” | The authors would like to thank the reviewer for the comment. The sentence has been amended accordingly | Synopsis. Line  67 | 3 |
| 12. | I assume these study sites are in Malaysia but I have no idea what part of Malaysia | The authors would like to thank the reviewer for the comment. The information about the location has been added accordingly | Introduction. Line  91-98  Methodology. Line  117 | 4  5 |
| 13. | 114bp is very small and problematic given that this is the sequence used for the phylogenetic analysis. | We agreed with the reviewer about the setbacks of short primer length. In this study, however, the first round of rt-PCR was still targeting a specific but broader WNV region which was 437 bp. The second round used shorter sequences to have more efficient annealing of the targeted gene. | Methodology. Line  188 | 8 |
| 14. | What dichotomous key was used to ID the mosquitoes morphologically? With no key mentioned and the heavy emphasis on mosquito communities, with specific species, how confident are the authors in the identifications? Almost appears as though molecular species ID would have helped in this study. | We believe using molecular methods is one of the best options for mosquito species determination. Having said that, the morphological determination used in this study has been tutored and verified by the experienced entomologist, who is also the writer of the ‘Illustrated Key Mosquito in Peninsular Malaysia’ book [17] (Jefferry et al., 2012). | Methodology. Line  162 | 7 |
| 15. | If these primers come from a previous publication, that can be indicated here in the text. | The authors would like to thank the reviewer for the comment. The detail has been tabulated in Table 1. | Methodology. Line  188 | 8 |
| 16. | With WNV circulating around the world for multiple decades and a vast body of research has been conducted along with the development of molecular diagnostic tools, why did this study need to design unpublished primers? | The authors would like to thank the reviewer for the comment. The first round of primer used was from a previous publication. But for the detection of WNV in mosquitoes we modified the procedure by adding one more round of PCR but still targeting the same region of PCR round 2, primarily to have more efficient annealing of the targeted gene in the small arthropod-like mosquito. | Methodology. Line  195 | 8 |
| 17. | I am having trouble finding any details in the methods or results regarding trap effort. How many trap locations were sampled and how many nights? | The authors would like to thank the reviewer for the comment. The information has been added as suggested by the reviewer. | Methodology. Line  133 | 6 |
| 18. | You are testing for virus in pooled mosquitoes so it is fine to present the percent of the pools that were infected but you should also consider pool size of those pos pools by calculating the WNV minimum infection rate (usually presented as XX per 1,000 mosquitoes). | The authors would like to thank the reviewer for the comment. The MIR analysis has been added as suggested by the reviewer. | Methodology. Line  218 | 9 |
| 19. | You need to be cautious claiming Culex are the dominant mosquito found in these sites given that you only had one sampling tool, and different mosquito species and physiological states are attracted to different trap types. | The authors would like to thank the reviewer for the comment. We agreed with the comment and the diversity section has been deleted. | - | - |
| 20. | Actually qPCR would be even more sensitive than nested PCR. I assume you didn’t have a real-time thermocycler, otherwise qPCR would have been advantageous for screening mosquito pools for virus and then a conventional PCR of a larger gene target could have been used for Sanger sequencing. | The authors would like to thank the reviewer for the understanding. Not using qPCR analysis is the limitation we have due to insufficient budget. We agreed that qPCR will have main advantage to screen the WNV in the mosquito pools. As we aware that the limitations of gel-based RT-PCR, we have included partial sequencing as our further quality control of the RT-PCR reaction. The positive pools from this study has been proceeded for full genome sequencing using the NGS approach. | - | - |
| 21. | All your phylogenetic analyses appear to be based on a very small gene fragment so reporting the percent similarity to other sequences could be mis-leading. | The authors would like to thank the reviewer for the comment. In our study, we believed the mis-leading concerns had been avoided by using two sets of primers targeting the conserved regions of WNV and had been confirmed by BLAST search as well. | - | - |
| 22. | Table 1. If the second round of primers was developed as part of the current study, then that column entry can say ‘this study’ instead of the ‘-‘. | The authors would like to thank the reviewer for the comment. The sentence has been amended accordingly. | Methodology. Line  188 | 8 |
| 23. | Table 4 could be removed with the data presented in the text. | The authors would like to thank the reviewer for the comment. The table has been removed accordingly. | - | - |
