# Supplementary material for: Circulation of West Nile virus in mosquitoes approximate to the migratory bird stopover in West Coast Malaysia
Source: PLoS Negl Trop Dis. 2023 Apr 6;17(4):e0011255. doi: 10.1371/journal.pntd.0011255 (PMC10112790; doi:10.1371/journal.pntd.0011255)
Supplement: Attachment — Submitted filename: Highlighted Version_Revised Manuscript PNTD-D-22-00829.rev1.docx [file pntd.0011255.s005.docx]

***Circulation of West Nile virus in mosquitoes approximate to the migratory bird stopover in West Coast Malaysia***

Jafar Ali Natasha^1^, Abd Rahaman Yasmin^1,2^*, Reuben Sunil Kumar Sharma^3^, Saulol Hamid Nur-Fazila^3^, Md Isa Nur Mahiza^3^, Siti Suri Arshad^3^, Hussni Omar Mohammed^4^, Kiven Kumar^5^, Shih Keng Loong^6^, Mohd Kharip Shah Ahmad Khusaini^7^

^1^Department of Veterinary Laboratory Diagnostics, Faculty of Veterinary Medicine, Universiti Putra Malaysia, 43400 UPM Serdang, Selangor, Malaysia

^2^Laboratory of Vaccines and Biomolecules, Institute of Bioscience, Universiti Putra Malaysia, 43400 UPM Serdang, Selangor, Malaysia

^3^Department of Veterinary Pathology and Microbiology, Faculty of Veterinary Medicine, Universiti Putra Malaysia, 43400 UPM Serdang, Selangor, Malaysia

^4^Department of Population Medicine and Diagnostic Sciences, Cornell University, Ithaca, New York, USA

^5^Department of Pathology, Faculty of Medicine and Health Sciences, Universiti Putra Malaysia, 43400 UPM, Serdang, Selangor, Malaysia

^6^Tropical Infectious Diseases Research & Education Centre, Higher Institution Centre of Excellence, University of Malaya, 50603, Kuala Lumpur, Malaysia

^7^Department of Conservation of Biodiversity of Wildlife and National Park Malaysia, Jalan Cheras, 56100, Kuala Lumpur, Malaysia

*Corresponding author:

E-mail: noryasmin@upm.edu.my (ARY)

**Abstract**

Being a tropical country with a conducive environment for mosquitoes, mosquito-borne illnesses such as dengue, chikungunya, lymphatic filariasis, malaria, and Japanese encephalitis are prevalent in Malaysia. Recent studies reported asymptomatic infection of West Nile virus (WNV) in animals and humans, but none of the studies included mosquitoes, except for one report made half a century ago. Considering the scarcity of information, our study sampled mosquitoes near migratory bird stopover wetland areas of West Coast Malaysia located in the Kuala Gula Bird Sanctuary and Kapar Energy Venture, during the southward migration period in October 2017 and September 2018. Our previous publication reported that migratory birds were positive for WNV antibody and RNA. Using a nested RT-PCR analysis, WNV RNA was detected in 35 (12.8%) out of 285 mosquito pools consisting of 2,635 mosquitoes, most of which were *Culex* spp. (species). Sanger sequencing and phylogenetic analysis revealed that the sequences grouped within lineage 2 and shared 90.12%–97.01% similarity with sequences found locally as well as those from Africa, Germany, Romania, Italy, and Israel. Evidence of WNV in the mosquitoes substantiates the need for continued surveillance of WNV in Malaysia.

**Author Summary**

West Nile virus (WNV) is considered to be one of the neglected diseases in Malaysia as most vector-borne febrile illnesses in humans are predominantly diagnosed as dengue-like illnesses. Nevertheless, WNV might be an addional virus causing the mosquito-borne disease in Malaysia based on data from previous studies. Our group previously identified the presence of WNV RNA and antibodies in the migratory and resident birds, macaques, bats and swine. However, there is no current data that could confirm the presence of WNV in the vector responsible for transmission of WNV to a wide range of hosts. Using molecular analysis, WNV RNA was detected from *Culex tritaeniorhynchus*, *Culex vishnui*, *Culex pseudovishnui*, *Culex gelidus*, *Armigeres subalbatus*, and *Coquillettidia* spp. Additionally, the sequence of WNV RNA was similar with the WNV lineage isolated from wild birds at the same location. These findings confirm the presence of an enzootic cycle of WNV in Malaysia and the risk for spill-over to humans and mammals.

**Introduction**

The abundance of mosquito vectors combined with movement of migrating birds across the globe has exacerbated the dynamics of West Nile virus (WNV) transmission [1]. WNV-infected birds particularly migrating passerine species are known to be a functional dispersal vehicle for the virus during long flying distances to the area where sufficient competent mosquitoes are available [2,3]. Mosquitoes such as *Culex* species acquire WNV from birds during blood feeding before spreading it to the native naïve birds and other susceptible vertebrate hosts [4]. Zoonotic implication of the amplifier and vector interaction to the dead-end host such as humans increase the rate of emerging and re-emerging diseases globally [5].

Current worldwide climate change and global warming phenomenon further contribute to the increment of climate-sensitive mosquito-borne infectious diseases, as increases in average temperature create an ideal environment for mosquito breeding and survival [6]. Malaysia, a tropical country with hot and humid weather together with inconsistent rainfall patterns, has an abundance of mosquito species along with high prevalence of mosquito-borne diseases every year such as endemic dengue and sporadic Japanese encephalitis and chikungunya outbreaks [7]. Thus far, there were no clinical cases of WNV in humans or animals occurring in the region. Differential diagnosis for febrile-like illness in humans has always been associated with dengue fever, and occasionally Japanese encephalitis if coupled with encephalitis syndrome. WNV, however, seems to not be in the differential list leaving the actual prevalence of infection unknown.

Historically, the first detection of WNV in Malaysia was from Sarawak, a Borneo state located in West Malaysia, during 1960 when it was isolated from *Culex pseudovishnui* [8]. In addition, WNV RNA and/or antibodies were discovered in multiple species, including companion birds, water birds, migratory birds, swine, bats, and macaques as well as in humans [9-12]. However, the current status of WNV in mosquitoes, in Peninsular Malaysia is lacking. Identifying possible vector species and sites that have a greater risk of WNV transmission are essential to plan for the prevention of WNV outbreaks in future.

The current study was carried out inKuala Gula Bird Sanctuary, Perak State, and Kapar Energy Venture, Selangor State. Both locations are situated near the Straits of Malacca from where WNV RNA and antibodies previously were detected in migratory and resident water birds [9]. Some of the mosquitoes reported here were collected simultaneously with that previous study; however, additional time was needed for the taxonomic identification of the specimens before virus detection, so the data were not presented together. The discovery of WNV in mosquito populations at these locations is a critical step to understand WNV circulation dynamics in the region.

**Methods**

**Sampling consent and approval**

A study approval from Institutional Animal Care and Use Committee (IACUC) was received from Universiti Putra Malaysia under reference number UPM/IACUC/AUP NO: R043/17. The approval from the Department of Wildlife and National Parks (DWNP), Peninsular Malaysia, was granted, with the research permit number JPHL&TN(IP):100-6/1/14 to collect mosquitoes from the wild bird landing areas.

**Study design and sampling location**

A cross-sectional study design was conducted near wetland areas located at the Kuala Gula Bird Sanctuary in Perak state (4.94° N latitude and 100.49° E longitude) and Kapar Energy Venture in the Selangor State (3.12° N latitude and 101.32° E longitude). The study location map was prepared using Natural Earth website: https://www.naturalearthdata.com/downloads/ (Fig 1).

**Fig 1. Sampling sites for mosquito collection.** The wild bird’s migration sites and stopover in Malaysia used as sampling location of mosquitoes in this study [marked with orange pin].

Both sites are facing the Straits of Malacca, a frequent stopover spot for migratory birds in Malaysia during southward and northward migration periods [13]. Kuala Gula Bird Sanctuary supports a large area of mangrove swamps and river estuary making it a renowned fishing village which is rich with small fish, mud crabs and prawns [9, 14]. Kapar Energy Venture thermal plant is located on intertidal mudflats containing an ash pond and is located around 3 to 5 km away from the nearest residential area and fishing pier [13]. Upon the migratory bird arrival, routine ringing (leg banding) was regularly conducted by the staff of the Department of Wildlife and National Parks (DWNP) and the sampling of the mosquitoes in this study were conducted in collaboration with the DWNP. Mosquitoes were collected based on convenient sampling with two sets of traps operated for two consecutive nights from October 17 to October 19, 2017, at Kuala Gula Bird Sanctuary and between September 12 to 14, 2018 at Kapar Energy Venture. Both locations support large numbers of migratory birds and provide suitable habitat for mosquito production. During sampling, both sites experienced monsoon season weather patterns whereby the rainfall was higher than normal period coupled with thunderstorms as alerted by the official website of Malaysian Meteorological Department.

**Mosquito collection**

Two traps were deployed approximately 200 to 500 meters from wild bird mist net trapping and resident bird roosting and breeding area (S1 Fig). Temperature was obtained from the official website of the Malaysian Meteorological Department (<https://www.met.gov.my/>), the temperature was 29.2°C and 22.0°C in Kuala Gula and Kapar, respectively, while the trapswere deployed [7] . Two Miniature CDC Light Traps Model 512 with a UV light (John W. Hock Company, USA) were suspended at 1.5 m height and baited with dry ice (Linde, Malaysia). The traps were operated overnight from 1800h to 0700h. The collecting bags containing mosquitoes were placed in an icebox and covered with a damp cloth as suggested by Coleman et al. [15] to keep the collected mosquitoes alive. Upon reaching the laboratory, the collecting bags were stored at −80°C (Sanyo, Japan) at the Laboratory of Virology, Faculty of Veterinary Medicine, Universiti Putra Malaysia, for later mosquito identification and analysis.

**Mosquito identification and analysis**

**Mosquito identification**

Mosquito identification was done on a chill table using taxonomic keys and morphological characteristics as described by Jeffery et al. [17] and Jourdain et al. [18]. Once identified mosquitoes were pooled by genus, sex, date, and collection site in a range of one to ten (1–10) mosquitoes per pool as suggested by Patsuola et al. [20]. Pools were stored at –80°C (Sanyo, Japan) in the Laboratory of Virology, Faculty of Veterinary Medicine, Universiti Putra Malaysia for the subsequent RNA extraction procedure.

**Molecular analysis**

**Mosquito homogenization**

After adding 500 ul of PBS, pooled mosquito samples were homogenised under 805 × *g* using a battery-operated pellet pestle (Sigma, USA) with an autoclavable plastic pestle (Biobasic, Canada) in a container filled with crushed ice until the mosquito was fully macerated. All homogenization was done in a Class 2 Biosafey Cabinet (ESCO, Singapore).

**Nested Reverse Transcriptase – Polymerase Chain Reaction (Nested RT-PCR)**

RNA was extrtacted from thehomogenised poolsusing Trizol (Bioline, UK), according to the manufacturer’s protocol. A nested reverse transcriptase-polymerase chain reaction (nested RT-PCR) assay was conducted to detect WNV. Two sets of primers targeting a conserved region of WNV, namely capsid and pre-membrane gene regions, were used (Table 1). A synthetic plasmid containing the WNV capsid and pre-membrane regions was used as the positive control [9].

**Table 1. Sequences of Primers Used in This Study**

|  | Primer | Sequence (5’ – 3’) | Amplicon Size (bp) | Reference |
| --- | --- | --- | --- | --- |
| First Round | Forward | CCAATACGTTTCGTGTTGG | 437 | [9] |
|  | Reverse | GGAAATGACCCTGAAGACAT |  |  |
| Second Round^a^ | Forward | GCTGGATCGATGGAGAGGTG | 114 | this study. |
|  | Reverse | CGGCGGAGCTCAAAACAAAA |  |  |

^a^The second-round primers were designed based on the reference strain WNV-1/Culex/USA/37030146/2003 (Accession no. KX547617) from Lineage 1.

In this study, the second round primer set was designed using the NCBI primer designing tool (<https://www.ncbi.nlm.nih.gov/tools/primer-blast/>) based on sequences from the WNV strain WNV-1/Culex/USA/37030146/2003 (Accession no. KX547617). A pair of primers were designed to flank the RT-PCR product and were amplified using primers previously reported by Najwa et al. (2020), resulting in a PCR product with the expected amplicon size of 114 bp. The first-round protocol was performed under the cycling condition as follows: reverse transcription at 95°C for 20 min, polymerase activation at 95°C for 1 min, 40 cycles of denaturation at 95°C for 10 s, annealing at 52°C for 10 s, and extension at 72°C for 30 s, which proceeded to the final extension at 72°C for 5 min and on hold for infinity at 10°C..The second round PCR was as follows: initial denaturation at 95°C for 1 min, 30 cycles of denaturation at 95°C for 15 s, annealing at 52°C for 15 s and extension at 72°C for 10 s.

**Phylogenetic analysis**

All positive amplicons from the nested RT-PCR assay were subjected to DNA sequencing. The nucleotide sequences (S1 Table) of the WNV-positive samples were subjected to Basic Local Alignment Search Tool (BLAST) analyses to determine the similarity of the amplicons compared to WNV strains from other regions. Sequences then were aligned using the Multiple Sequence Alignment (MAFFT) software version 7, using 52 WNV reference sequences (S2 Table) and were compared to positive sequences from this study and representative WNV sequences from wild birds from the same study sites. The phylogenetic tree was constructed using the maximum likelihood method employing the Jukes-Cantor model in the Molecular Evolutionary Genetics Analysis (MEGA) 7 software. A bootstrapped confidence interval of 1,000 replicates was set. The Newick file was created, and the tree was viewed and edited using the FigTree.v1.4.4.

**Minimum infection rate**

The positive mosquito pools were subjected to Minimum Infection Rate (MIR) calculation by using the formula equation below [21]:

$$Minimum Infection Rate \left( MIR \right)= \frac{Number of positive pools}{Total number of mosquitoes tested} \times1000$$

The MIR was further analysed with Chi-Square test with Statistical Package for the Social Sciences (SPSS), Version 25 (IBM, New York) to compare the MIR between mosquito species. The statistical analysis was applied with alpha level 0.05 at 95 % significant confidence interval.

**Results**

**Mosquito identification**

Overall, 2,635 mosquitoes comprising 11 genera and 19 species were collected (S2 Fig). Of these, 2,621 were females. Two *Culex* subgenera were identified, namely, *Lophoceraomyia* and *Culex,*which included the reputed arbovirus vectors*Culex tritaeniorhynchus*, *Culex vishnui*, *Culex pseudovishnui*, *Culex quinquefasciatus*, and *Culex gelidusCulex tritaeniorhynchus* was identified by the dark to black occipital scales, with the ventral part of the proboscis outstretching proximally, differentiating it from the other *Culex* species.

The morphology classification for the *Cx. vishnui* species is the dark brown vertex scale on top of the head, with parts of the scutum covered with dark brown scales. In contrast, the taxonomic key of *Cx. pseudovishnui* is the light to brown scales on the scutum and pale scales on the vertex. The apparent feature of *Cx. gelidus* is the scutum, located on the dorsal part of the thorax that is covered with white scales up to the prescutellar area at the ventral of the scutum. In comparison, *Cx. quinquefasciatus* has striped colour on the abdomen. The basal area is paler than the apical area, accompanied by the absence of a pale and dark pattern in the mesokatepisternum and mesepimeron site on the lateral part of the thorax.

**Detection of WNV RNA**

WNV RNA was detected in 35 out of 285 (12.28%) mosquito pools (Table 3, Fig 2). The positive WNV pools were detected from six different species: *Cx. tritaeniorhynchus* (24 pools), *Cx. vishnui* (6 pools), *Cx. pseudovishnui* (2 pools), *Cx. gelidus* (1 pool), *Armigeres subalbatus* (1 pool), and *Coquillettidia* spp. (Table 3). All the positive mosquito pools comprised female mosquitoesOne of 24 positive mosquito pools of *Cx. tritaeniorhynchus* was found in Kuala Gula Bird Sanctuary, Perak, while the remaining positive mosquito pools were found in Kapar Energy Venture, Selangor. Only one pool was from Oct 2017, while the remaining 34 pools were from Sept 2018.

**Fig 2. Bands of positive mosquito samples from the gel electrophoresis procedure**. The expected target size is 114 bp, targeting capsid and pre-membrane genes of the WNV. The gel was prepared with 1.5% agarose gel electrophoresis (w/v). Key: L: 100 bp size ladder; +ve ctrl: negative control (deionised distilled water); +ve ctrl: positive control (WNV plasmid); S1–S5: pooled samples of female *Cx. vishnui* mosquitoes.

**DNA Sequencing**

**Partial DNA Sequencing and BLAST**

Partial sequences (forward and reverse) of positive amplicons from this study were combined using the BioEdit software (version 7.2.5.0) after generating the reverse complement strands. Positive WNV RNA pools were confirmed bypartial DNA sequencing analysis using BLAST tools. The DNA from viruses this study had a high similarity with Malaysian strains from Perak and Selangor (wild bird), ranging between 97.01% and 92.01%, agreeing with sequences fromSouth African strains with 92.59%; Germany (MH986055), Romania (KJ934710), Uganda (KY523178), and Israel (KC131128) strains with 91.36% homology; and Italian strains (MN939557 and MN939564) with 90.12% homology.

**Phylogenetic analysis**

A phylogenetic tree constructed using the maximum likelihood method with the Jukes-Cantor model showed evolutionary relationships of strains with WNV from this study. The positives grouped with WNV strains from the WNV lineage 2 (Fig 3). All positive amplicons from this study share the same branch, which diverged from other lineage 2 WNV nucleic acids.

**Fig 3. Phylogenetic analysis of WNV detected in the mosquito pools in Malaysia with other global WNV.** The analysis includes 52 WNV nucleotide sequences from other countries, 4 WNV nucleotides sequences of wild birds from Malaysia (presented in the blue box) and 35 sequences from this study (presented in the red box). The tree branches are colored according to the proposed WNV lineages; green: Lineage 1; orange: Lineage 2; other lineages (3, 4a, 4c,5 and 6): purple. The tree was constructed using the maximum likelihood method with the Jukes-Cantor model with 1000 bootstrap replicate. Branches are scaled bootstraps.

**Minimum Infection Rate**

Overall, the minimum infection rate (MIR) was 13.3 infected females per 1,000 tested. Species-wise, *Cx. tritaeniorhynchus* had the highest MIR, 9.11, followed by *Cx. vishnui* (2.28) and *Cx. pseudovishnui* (0.76). Meanwhile, *Cx. gelidus, Armigeres subalbatus, Coquillettidia* spp. presented with a similar MIR value of 0.38. A Chi-square test showed no significant difference among MIR values for mosquito species(χ^2^ = 20.40, p = 0.254)

**Table 3. Mosquito Distribution According to Location, Genus, Species and Pool Number**

| Habitat | Genus | Species | Sex | | Total Mosquito by Species | Number of mosquitoes per pool | Number of pools | Positive WNV RNA pools | Accession Number |
| --- | --- | --- | --- | --- | --- | --- | --- | --- | --- |
|  |  |  | M | F |  |  |  |  |  |
| Kuala Gula Bird Sanctuary, Perak | *Aedeomyia (Aedeomyia)* | *catastica* |  | 1 | 1 | 1 | 1 | - |  |
|  | *Aedes (Aedes)* | *albopictus* |  | 6 | 6 | 6 | 1 | - |  |
|  | *Armigeres (Armigeres)* | *kesseli* |  | 1 | 1 | 1 | 1 | - |  |
|  | *Armigeres (Armigeres)* | *maximus* |  | 1 | 1 | 1 | 1 | - |  |
|  | *Culex (Culex)* | *gelidus* |  | 36 | 36 | 10 | 4 | - |  |
|  |  | *pseudovishnui* | 4 | 84 | 88 | 10 | 10 | - |  |
|  |  | *tritaeniorhynchus* | 2 | 994 | 996 | 10 | 101 | 1 | ON809701 |
|  |  | *quinquefasciatus* | 2 | 2 | 4 | 2 | 2 | - |  |
|  |  | *vishnui* | 5 | 274 | 279 | 10 | 29 | - |  |
|  | *Culex (Lophoceraomyia*) | *spp.* | 1 | 1 | 2 | 1 | 2 | - |  |
|  | *Mansonia (Mansonioides)* | *annulifera* |  | 1 | 1 | 1 | 1 | - |  |
|  | *Uranotaenia* | *spp.* |  | 1 | 1 | 1 | 1 | - |  |
|  | *Verralina (Verralina)* | *butleri* |  | 1 | 1 | 1 | 1 | - |  |
| Kapar Energy Venture, Selangor | *Aedes (Aedes)* | *albopictus* |  | 2 | 2 | 2 | 1 | - |  |
|  | *Anopheles (Cellia)* | *spp.* |  | 1 | 1 | 1 | 1 | - |  |
|  | *Armigeres (Armigeres)* | *subalbatus* |  | 2 | 2 | 2 | 1 | 1 | TBR |
|  | *Coquillettidia (Coquillettidia)* | spp. |  | 1 | 1 | 1 | 1 | 1 | ON815484 |
|  | *Culex (Culex)* | *gelidus* |  | 3 | 3 | 3 | 1 | 1 | ON815483 |
|  |  | *pseudovishnui* |  | 154 | 154 | 10 | 16 | 2 | ON815482 |
|  |  | *tritaeniorhynchus* |  | 550 | 550 | 10 | 55 | 23 | OP580528 |
|  |  | *vishnui* |  | 480 | 480 | 10 | 48 | 6 | OM945733; ON809702 |
|  | *Culex (Lophoceraomyia* | *spp.* |  | 1 | 1 | 1 | 1 | - |  |
|  |  | *spp.* |  | 18 | 18 | 10 | 2 | - |  |
|  | *Heizmannia (Heizmannia)* | *spp.* |  | 1 | 1 | 1 | 1 | - |  |
|  | *Topomyia* | *spp.* |  | 1 | 1 | 1 | 1 | - |  |
|  | *Verralina (Verralina)* | *butleri* |  | 4 | 4 | 4 | 1 | - |  |
| Total | | | **14** | **2621** | **2635** |  | **285** | **35** |  |

M is indicated as male mosquitoes while F is indicated as female mosquitoes. TBR is indicated as to be retrieved. Maximum number of pools per species is 10 while the minimum is 1.

**Discussion**

Along the west coast of Peninsular Malaysia, Kuala Gula Bird Sanctuary and Kapar Energy Venture are the typical areas where migratory birds stopover from northern and southern flyways in Malaysia [13]. This area also supports a wide variety of water birds that reputedly are the principal reservoirs WNV and JEV [22, 23]. Najwa et al. [9], who conducted the sampling of wild birds’ sera and oropharyngeal swabs in the same locations as this study, found that WNV antibody was detected in 29/155 birds (18.71%), while WNV RNA was detected from 16/105 birds (15.2%). These findings indicate high levels of virus transmission.

Nested RT-PCR was performed to detect WNV RNA in the homogenised mosquitoes by targeting the conserved regions of WNV between the capsid and pre-membrane protein regions. This type of PCR is highly sensitive as opposed to conventional PCR, which previously detected the presence of WNV from lineage 1 and 2 [38-40]. Overall, 35 out of the 285 pools mosquitoes were positive for WNV RNA (12.28%) and *Cx. tritaeniorhynchus* yielded the highest MIR. Presence of WNV in field collected *Cx. tritaeniorhynchus* from 2014 to 2015 was considered a breakthrough finding in Assam, India with a 0.36 MIR [39]. An experimental infection study in China by Jiang et al. [41] revealed that *Cx.* *tritaeniorhynchus* was among thecompetent vectors of WNV. Similarly, Akhter et al. [42] showd that *Cx. tritaeniorhynchus* required the lowest median WNV infective dose as compared to other tested mosquito species from Pakistan. These results were interesting from a host blood meal preference perspective, because *Cx. tritaeniorhynchus* typically feeds more frequently on large mammals than birds [43,44].Fall et al [44] reported that *Cx. tritaeniorhynchus* densities near to pigeon habitat increased dramatically following the arrival of migratory birds in the region and then declined over time . The high infection prevalence of WNV in this mosquito species supportedits role as a vector of WNV during migratory bird arrival and rainy season.

The partial sequencing analysis revealed that the lineage 2 strains detected during our study had approximately 97.01% similarity with WNV from wild birds at the same study site and from South African and American crows. These studies collectively indicated that WNV lineage 2 has been circulating in Malaysia and implicated migratory birds in viral introduction.. The intercontinental relatedness of the WNV isolates from Malaysia may be explained by whole genome sequencing, or at the very least the hypervariable domain.

Some of the sampling of mosquitoes from our study was conducted simultaneously with wild birds sampling in Kuala Gula and Kapar. Our previous data reported that birds from *Ardeidae*, *Charadriidae* and *Scolopacidae* families were shedding WNV RNA and were seropositive to WNV [9]. In addition, the JEV antibody was also detected in wild birds in this area [52]. Our study confirmed the presence of an enzootic WNV cycle of involving mosquitoes and birds in Peninsular Malaysia . Further studies are warranted to investigate the potential zoonotic link of WNV in the human populations living near the study sites.

**Conclusion**

In summary, findings from the current study indicated that WNV was present in most *Culex* species collected, as demonstrated by the detection of WNV RNA from the nested PCR analysis. WNV RNA from mosquitoes and birds originated from the same lineage 2, indicating that this lineage circulates in Malaysia. A further entomological investigation is required to describe the feeding and breeding preferences, virus surveillance, and mutations. Programmes targeting the prevention of transmission of WNV through vector control, public awareness, and biosurveillance of pathogens in wild birds are being implemented via one health initiative among different authorities. This study was in line with Malaysia Strategy for Emerging Diseases and Public Health Emergencies (MYSED) II (2017–2021) under the Ministry of Health, which prioritised enhancing regional capability to rapidly and accurately survey, detect, diagnose, and report outbreaks of pathogens and diseases of public health security concern.

**Acknowledgements**

We would like to thank the Department of Wildlife and National Parks (DWNP), Peninsular Malaysia, for the sampling approval. The greatest appreciation is attributed to the late Mr John Jeffery, the writer of entomology books, for his guidance and help with the mosquito identifications.

**References**

1. Maquart M, Boyer S, Rakotoharinome VM, Ravaomanana J, Tantely ML, Heraud JM, et al. High prevalence of West Nile virus in domestic birds and detection in 2 new mosquito species in Madagascar. PLoS One. 2016; 11: e0147589.
2. Owen J, Moore F, Panella N, Edwards E, Bru R, Hughes M, et al. Migrating birds as dispersal vehicles for West Nile virus. Ecohealth. 2006; 3:79-85.
3. Ezenwa VO, Godsey MS, King RJ, Guptill SC. Avian diversity and West Nile virus: testing associations between biodiversity and infectious disease risk. Proc Biol Sci. 2006; 273:109-117.
4. Farajollahi A, Fonseca DM, Kramer LD, Kilpatrick AM. “Bird biting” mosquitoes and human disease: a review of the role of Culex pipiens complex mosquitoes in epidemiology. Infect Genet Evol. 2011; 11: 1577-1585.
5. Wu X, Lu Y, Zhou S, Chen L, Xu B. Impact of climate change on human infectious diseases: empirical evidence and human adaptation. Environ Int. 2016; 86: 14-23.
6. Filho WL, Scheday S, Boenecke J, Gogoi A, Maharaj A, Korovou S. Climate Change, Health and Mosquito-Borne Diseases: Trends and Implications to the Pacific Region. Int J Environ Res Public Health. 2019; 16:5114.
7. General Climate in Malaysia. [cited 20 June 2022]. In: Official Website of Malaysian Meterological Malaysia [Internet]. Selangor 2021. Available from: <https://www.met.gov.my/>
8. Bowen ETW, Simpson DIH, Platt GS, Way HJ, Smith CG, Ching CY, et al. Arbovirus infections in Sarawak: the isolation of Kunjin virus from mosquitoes of the Culex pseudovishnui group. Ann Trop Med Parasitol. 1970; 64: 263-268.
9. Ain-Najwa MY, Yasmin AR, Omar AR, Arshad SS, Abu J, Mohammed HO, et al. Evidence of West Nile virus infection in migratory and resident wild birds in west coast of peninsular Malaysia. One Health. 2020; 10: 100134.
10. Ain-Najwa MY, Yasmin AR, Arshad SS, Omar AR, Abu J, Kumar K, et al. Exposure to zoonotic West Nile virus in long-tailed macaques and bats in peninsular Malaysia. Animals. 2020; 10: 2367.
11. Mohammed MN, Yasmin AR, Ramanoon SZ, Arshad SS, Bande F, Mohammed HO. Serological evidence of West Nile viral infection in archived swine serum samples from peninsular Malaysia. J Vet Sci. 2021; 22: e29.
12. Marlina S, Radzi SF, Lani R, Sieng KC, Rahim NF, Hassan H, et al. Seroprevalence screening for the West Nile virus in Malaysia's Orang Asli population. Parasit Vectors. 2014; 17: 7:59.
13. Bakewell D. Kapar Power Station Waterbird Report 2008. Kuala Lumpur, Malaysia: MNS-Bird Conservation Council. MNS-BCC Waterbirds Group Report No.1. 2009 [Cited 2022 June 20]. Available from: <http://mnswetland.weebly.com/uploads/3/1/8/0/31801977/kapar_power_station_waterbird_report_2008_final.pdf>.
14. Rahman F, Ismail A, Shohaimi S, Omar H, Hussin MZ. Foraging activity of the re-introduced milky storks (*Mycteria cinerea*) in Kuala Gula Bird Sanctuary, Perak, Malaysia. Pertanika J Trop Agric Sci. 2020; 43: 217-229.
15. Coleman WB, Tsongalis GJ. Molecular Pathology: The molecular basis of human diseases. In: Williams ES, Silverman LM, editors. Molecular diagnosis of human disease. 2018. pp. 691-707.
16. Becker N, Zgomba M, Boase C, Madon M, Dahl C, Kaiser A. Mosquito and their control. 2nd ed. New York: Springer Heidelberg Dordrecht; 2010.
17. Jeffery J, Rohela M, Muslimin M, Abdul Aziz SMN, Jamaiah I, Kumar S, et al. Illustrated keys: some mosquitoes of peninsula Malaysia, Malaysia. 1st ed. Malaysia: University of Malaya Press; 2012.
18. Jourdain F, Picard M, Sulesco T, Haddad N, Harrat Z, Sawalha SS, et al. Identification of mosquitoes (Diptera: Culicidae): an external quality assessment of medical entomology laboratories in the MediLabSecure Network. Parasit vector. 2018; 11: 553.
19. Armstrong PM, Andreadis TG, Finan SL, Shepard JJ, Thomas MC. Detection of Infectious Virus from Field-collected Mosquitoes by Vero Cell Culture Assay. JoVE. 2011; 52.
20. Patsoula E, Beleri S, Tegos N, Mkrtsian R, Vakali A, Pervanidou D. Entomological data and detection of West Nile virus in mosquitoes in Greece (2014–2016), before disease re-emergence in 2017. Vector Borne Zoonotic Dis. 2020; 20: 60-70.
21. Condotta SA, Hunter FF, Bidochka MJ. West Nile virus infection rates in pooled and individual mosquito samples. Vector Borne Zoonotic Dis. 2004; 4: 198-203.
22. Hamer GL, Walker ED, Brawn JD, Loss SR, Ruiz MO, Goldberg TL, et al. Rapid amplification of West Nile virus: the role of hatch-year birds. Vector Borne Zoonotic Dis. 2008; 8: 57-67.
23. Kuwata R, Hoshino K, Isawa H, Tsuda Y, Tajima S, Sasaki T, et al. Establishment and characterization of a cell line from the mosquito *Culex tritaeniorhynchus* (Diptera: Culicidae). In Vitro Cell Dev Biol Anim. 2012; 48: 369-376.
24. Anderson SL, Richards SL, Smartt CTA. A simple method for determining arbovirus transmission in mosquitoes. J Am Mosq Control Assoc. 2010; 26: 108-111.
25. González MA, Prosser SW, Hernández-Triana LM, Alarcón-Elbal PM, Goiri F, López S, et al. Avian feeding preferences of *Culex pipiens* and *Culiseta* spp. along an urban-to-wild gradient in northern Spain. Front Ecol Evol. 2020; 8: 568835.
26. Kauffman E, Payne A, Franke MA, Schmid MA, Harris E, Kramer LD. Rearing of *Culex* spp. and *Aedes* spp. mosquitoes. Bio Protoc. 2017; 7: e2542.
27. Kweka EJ, Baraka V, Mathias L, Mwang’onde B, Baraka G, Lyaruu L, et al. Ecology of *Aedes* mosquitoes, the major vectors of arboviruses in human population. In: Abelardo Falcón-Lezama J., Betancourt-Cravioto M., Tapia-Conyer R, editors. Dengue fever – a resilient threat in the face of innovation. London: IntechOpen; 2018. pp. 39-55.
28. Silver JB. Mosquito ecology: field sampling methods. 3rd ed. New York: Springer Heideberg Inchelhit; 2018.
29. Wolbachia-*Aedes* Mosquito Suppression Strategy. [cited 20 June 2022]. In: National Environment Agency [Internet]. Singapore. Available from: https://www.nea.gov.sg/corporate-functions/resources/research/wolbachia-aedes-mosquito-suppression-strategy/male-mosquitoes-do-not-bite
30. Dormont L, Mulatier M, Carrasco D, Cohuet, A. Mosquito attractants. J Chem Ecol. 2021; 47,: 351-393.
31. Boyer S, Tantely ML, Randriamaherijaona S, Andrianaivolambo L, Cardinale E. Mosquitoes sampling strategy for studying West Nile virus vectors in Madagascar: abundance, distribution and methods of catching in highrisk areas. Arch Inst Pasteur Madagascar. 2014; 71: 1-8.
32. Sriwichai P, Karl S, Samung Y, Sumruayphol S, Kiattibutr K, Payakkapol A, et al. Evaluation of CDC light traps for mosquito surveillance in a malaria endemic area on the Thai Myanmar border. Parasit vector. 2015; 8: 636.
33. Hutchinson RA, West PA, Lindsay SW. Suitability of two carbon dioxide-baited traps for mosquito surveillance in the United Kingdom. Bull Entomol Res. 2007; 97: 591-597.
34. Balenghien T, Vazeille M, Grandadam M, Schaffner F, Zeller H, Reiter P, et al. Vector competence of some French *Culex* and *Aedes* mosquitoes for West Nile virus. Vector Borne Zoonotic Dis. 2008; 8: 589-595.
35. Engler O, Savini G, Papa A, Figuerola J, Groschup MH, Kampen H, et al. European surveillance for West Nile virus in mosquito populations. Int J Environ Res Public Health. 2013; 10: 4869-4895.
36. Vogels CB, Göertz GP, Pijlman GP, Koenraadt CJ. Vector competence of European mosquitoes for West Nile virus. Emerg Microbes Infect. 2017; 6: e96.
37. Dale PER, Knight JM. Wetlands and mosquitoes: a review. Wetl Ecol Manag. 2008; 16: 255-276.
38. Ergunay K, Gunay F, Erisoz Kasapj O, Oter K, Gargari S, Karaoglu T, et al. Serological, molecular and entomological surveillance demonstrates widespread circulation of West Nile virus in Turkey. PLoS Negl Trop. Dis. 2014; 8: e3028.
39. Khan SA, Chowdhury P, Choudhury P, Dutta P. Detection of West Nile virus in six mosquito species in synchrony with seroconversion among sentinel chickens in India. Parasit Vector. 2017; 10: 13.
40. Shi PY, Kauffman EB, Ren P, Felton A, Tai JH, Dupuis AP, et al. High-throughput detection of West Nile virus RNA. J Clin Microbiol. 2001; 39: 1264-1271.
41. Jiang SF, Zhang YM, Guo XX, Dong YD, Xing D, Xue RD. Experimental studies on comparison of the potential vector competence of four species of Culex mosquitoes in China to transmit West Nile virus. J Med Entomol. 2010; 47: 788-790.
42. Akhter R, Hayes CG, Baqar S, Reisen WK. West Nile virus in Pakistan. III. Comparative vector capability of *Culex tritaeniorhynchus* and eight other species of mosquitoes. Trans R Soc Trop Med Hyg. 1982; 76: 449-453.
43. Reisen WK, Boreham PF. Host selection patterns of some Pakistan mosquitoes. Am J Trop Med Hyg. 1979; 28:408-421.
44. Fall AG, Diaïté A, Lancelot R, Tran A, Soti V, Etter E, et al. Feeding behaviour of potential vectors of West Nile virus in Senegal. Parasit Vectors. 2011; 8: 4:99
45. Mosquito Surveillance Software. [cited 20 September 2022] In: Centre for Disease Control and Prevention [Internet]. United States. vailable from: https://www.cdc.gov/westnile/resourcepages/mosqSurvSoft.html
46. Chakraborty S, Smith RL. Error associated with estimates of minimum infection rate for endemic West Nile virus in areas of low mosquito trap density. Sci Rep. 2019; 9: 19093.
47. Pérez-Ramírez E, Llorente F, Jiménez-Clavero MÁ. Experimental infections of wild birds with West Nile virus. Viruses. 2014; 6: 752-781.
48. Basari N, Aiman Syazwan H, Mohd Zairi Z, Nur Aida H. Larval distributions and breeding habitats of *Aedes aegypti* and *Ae. albopictus* in Kuala Terengganu. Trop Biomed. 2016; 33: 420-427.
49. Seidahmed OM, Eltahir EA. A sequence of flushing and drying of breeding habitats of *Aedes aegypti* (L.) prior to the low dengue season in Singapore. PLoS Negl Trop Dis. 2016; 10: e0004842.
50. Papa A, Danis K, Baka A, Bakas A, Dougas G, Lytras T, et al. Ongoing outbreak of West Nile virus infections in humans in Greece, July-August 2010. Euro Surveill. 2010; 15:19644
51. Benbetka S, Hachid A, Benallal KE, Benbetka C, Khaldi A, Bitam I, et al. First field evidence of infection of *Culex perexiguus* by West Nile virus in Sahara Oasis of Algeria. J Vector Borne Dis. 2018; 55: 305-309.
52. Kumar K, Arshad SS, Selvarajah GT, Abu J, Toung OP, Abba Y, et al. Prevalence and risk factors of Japanese encephalitis virus (JEV) in livestock and companion animal in high-risk areas in Malaysia. Trop Anim Health Prod. 2018; 50: 741-752.

**Supporting Information Captions**

**S1 Fig. CDC Light Trap setting baited with dry ice.**

**S2a Fig. Microscopic image on anterior view of the mosquito genera (a) *Aedeomyia*, (b) *Aedes*, (c) *Anopheles* and (d) *Armigeres.***

**S2b Fig. Microscopic image on lateral view of the mosquito genera (a) *Coquillettidia*, (b) *Culex*, (c) *Heizmannia* and (d) *Mansonia.***

**S2c Fig. Microscopic image on anterior view of the mosquito genera (a) *Topomyia*, (b) *Uranotaenia* and (c) *Verralina*.**

**S3 Fig. Microscopic image on lateral view of mosquito based on sex. Male is characterized by plumose antenna and long maxillary palp, while female is characterized by pilose antenna and short maxillary palp. Left: Male; Right: Female.**

**S1 Table. Partial DNA Sequence of Positive Isolates of WNV RNA from Mosquitoes**

**S2 Table. List of Reference strain of WNV used in this study.**
