# Supplementary material for: Circulation of West Nile virus in mosquitoes approximate to the migratory bird stopover in West Coast Malaysia
Source: PLoS Negl Trop Dis. 2023 Apr 6;17(4):e0011255. doi: 10.1371/journal.pntd.0011255 (PMC10112790; doi:10.1371/journal.pntd.0011255)
Supplement: Attachment — Submitted filename: Rebuttal Letter and Response to Reviewer Comments.docx [file pntd.0011255.s006.docx]

03^rd^ February 2023

Editor-in-Chief,

PLOS Neglected Tropical Disease,

Shaden Kamhawi,

Vector Molecular Biology,

National Institutes of Health,

Rockville, MD, USA

Dear Editor,

REVISED MANUSCRIPT FOR PUBLICATION

Enclosed herewith is a revised manuscript entitled “Circulation of West Nile Virus in Mosquitoes Approximate to the Migratory Bird Stopover in West Coast Malaysia” for publication in PLOS Neglected Tropical Disease.

We thank reviewers for suggestions and comments. We the feel that the reviewer's comments helped clarify and improve the manuscript. The response to the comments is attached. With the submission of this manuscript, I would like to undertake that the above-mentioned manuscript has not been published elsewhere, accepted for publication elsewhere, or under editorial review for publication elsewhere.

I hope the manuscript is in satisfactory condition and has the merit for publication in PLOS Neglected Tropical Disease.

Thank you in advance.

With best regards,

------------------------------------------------

Dr. Nor Yasmin Abd. Rahaman

Corresponding author

E-mail: [noryasmin@upm.edu.my](mailto:noryasmin@upm.edu.my)

**Response to Reviewers**

Dear Reviewers,

The authors have revised the manuscript accordingly and feel that the reviewer's comments helped clarify and improve the manuscript. Kindly please find our responses.

| **Section** | **Reviewer** | **Comments** | **Response** |
| --- | --- | --- | --- |
| Summary and General Comments | Reviewer 1 | The data shows that females from a several *Culex* species collected by CDC CO_2_ trap were infected with WNV L2 and that this RNA was similar to RNA extracted and sequenced from migratory birds collected at the same study sites.  This indicates that there is most likely an enzootic cycle established at these wetlands.  Recommendations for additional studies was well-taken. | - |
|  | Reviewer 3 | The manuscript "Circulation of West Nile virus in mosquitoes approximate to the migratory bird stopover in West Coast Malaysia" by Natasha et al reports an interesting study that aimed at evaluating the presence of WNV in mosquitoes that were sampled near to a migratory bird stopover. This is an interesting study and the manuscript is well-written. Important changes in the manuscript have been performed according to the considerations of the other reviewers. However, I still have some considerations and concerns about the study, as follows:  1. The species of the mosquitoes were not confirmed through a molecular test, which is concerning mainly because of the similarities between *Culex* species that may compromise the visual identification.  2. Did each mosquito pool comprise only male or female mosquitoes, or the mosquitoes were mixed regardless of sex in an aleatory way? This information should be clear in the manuscript.  3. Lines 401-2: "the involvement of mosquitoes as competent vectors". The fact that the mosquitoes are infected with the virus suggests but does not prove that the mosquitoes from the region are competent vectors to infect vertebrates, since the virus can perpetuate in a mosquito population through other mechanisms such as vertical transmission. Moreover, the vectorial competence varies between mosquitoes of the same species from different geographical areas. Therefore, I suggesting the rephrasing of this excerpt.  4. Lines 412-3: "This study suggests the possible role of Culex species as the main vector of WNV transmission in the region". Again, as I explained above in the point 3, the fact that the mosquitoes of a given species were found to be infected with the virus does not necessarily mean that they are the main vector of the WNV, because the other mosquito species with an inferior MIR could be more competent to transmit the virus through bite.  5.Furthermore, I suggest that future investigations evaluate mosquito larvae collected in that geographical area for investigating vertical transmission of the virus, which can be a mechanism that is favoring the perpetuation of the WNV in the mosquito populations. Moreover, it would be interesting to investigate the vectorial competence of the mosquitoes from that area, in order to establish the capacity of the mosquitoes from the region to infect vertebrates, since previous studies demonstrate that the transmission of arboviruses varies between mosquitoes from different geographical areas. | ***The authors would like to express great appreciation for the reviews and comments. The comments were taken into account seriously and amended accordingly.***  1. The authors would like to thank the reviewer for the comment and agrees that using molecular methods is one of the best options for mosquito species determination. Due to time and cost constrain, it was not included in the study. Having said that, the morphological determination used in this study has been tutored and verified by the experienced entomologist, who is also the writer of the ‘Illustrated Key Mosquito in Peninsular Malaysia’ book [17] (Jeffery et al., 2012).  2.Yes, the mosquitoes were also separated based on the sex  **[Line 160; pp 7].**  3. We agreed with the reviewer’s comment pertaining on the competent vector point of view. We have omitted that explanation.  4. Thank you, we have deleted that part accordingly and revised the explanation.  5. Thank you for the suggestion, we will definitely consider your valuable suggestion to expand this study and has added it in the future study. **[Line 344; pp 17].** |
|  | Reviewer 4 | The manuscript lays a lot of emphasis on the description of the methods and findings, but omits crucial sections on objectives, hypotheses, and limitations | The authors would like to express great appreciation for the revision and editing of the manuscript. Clear hypothesis, objectives and limitations have been added/amended throughout the manuscript as suggested by the reviewer. |
|  | Reviewer 5 | In the introduction: Line 86: The authors introduce the concepts of Malaysia having topic weather, as well as endemic dengue and sporadic JEV and CHIKV outbreaks.  They cite a source for the climate concepts but not for the endemic dengue or outbreaks of mosquito-borne viruses.  This would strengthen the argument for the need for surveillance of WNV in mosquitoes. | The reference has been added accordingly as suggested by the reviewer **[Line 83; pp 4].** |
| Method | Reviewer 1 | a. There was no succinct hypothesis stated or tested in this paper.  A previous study detected WNV RNA and antibody in migratory water birds and the current study was done to determine if these birds were able to infect local mosquito populations.  The work done established the presence of WNV RNA in mosquitoes collected at the sites of previous bird studies.  This indicated that the migrants may have actually been infected locally by mosquitoes??? Mosquitoes were collected by CDC traps -- Supplemental Figure 1 was not necessary.  b. Mosquito identification was done on chill tables and the specimen identifications were confirmed morphologically by an expert before homogenization and testing for virus.  Detailed descriptions of the species and associated Supplemental Figures 2 and 3 were not necessary.  Therefore, there were no voucher specimens saved.  c. The biggest methodological issue was the species identification of specimens pooled for virus.  Line 172 indicated specimens were pooled 'according to genus'?   It was unclear then how species-specific data was later presented in the results and Table 3????  ***[from track changes*]**  d*.* In Abstract - This disagrees with the Sep and Oct sampling intervals within the M&M section????  e. In author summary - Birds are animals?  f. In intro- This statement precludes that WNV does not persist in Malaysia?  g. In intro- Unclear? Reword sentence  h. What is this?  i.Delete figure.  j. Move to acknowledgements or include as author?  k. But Table 3 shows the number of each species within each pool? How could this be determined if pools included multiple species as implied her?  l. Since your group detected JEV antibody in birds, why didn’t you test these mosquitoes for JEV RNA  m. So you would not detect Lineage II? This WNV99 strain?  n. Delete photos of mosquitoes.  o. Delete this and the associated Figures. You indicated you followed published keys by Jeffery, and he helped verify the IDs. That is enough  p. Because mosquitoes were pooled by genus [see M&M section] how did you produce this species specific data????  q. Isolates are whole viruses in culture or frozen; you have DNA sequences from RT-PCR. Not the same.  r. Interesting as you positive controls were from Lineage 1?  s. Where is Table 2?  t. This is way too long and wordy. Discuss the principal findings of this study and not general background information such as trap efficiency, etc.  u. Aren’t these the same flyway used in both directions?  v. But these would infected with Lineage 1 | ***The authors would like to thank the reviewer for the comment.***  a. Hypothesis has been added as suggested by the reviewer **[Line 105; pp 5]**  - The supplementary figure 1 has been deleted.  b. The supplementary figures 2 and 3 have been deleted  c. The author apologizes for the confusion. The mosquitoes were identified up to species level and the sentences was amended accordingly **[Line 160; pp 7]**  ***[from track changes*]**  d. The sampling time information has been amended and aligned with methodology section **[Line 39; pp: 2]**  e. The statement has been changed to other vertebrates. **[Line 67; pp 3]**  f. In the Malaysian context, we have molecular and serological evidence of WNV presence in animals & vector mosquitoes. Most of these surveillance studies were conducted within a moderate time frame of up to 3 years and one limitation to this type of study is that the information on the detected pathogens is only captured within the particular time frame. In the case of WNV however, we believe that the virus persists in an avian-mosquito-wildlife/livestock cycle at low levels. We think that this is due to the migration season for birds that occurs only for several months in a year and the virus is maintained at very low levels of viraemia in susceptible animal hosts. And, this actually helps in the persistence of WNV in Malaysia.  g. The statement about *emerging and re-emerging* has been amended accordingly as suggested by the reviewer. **[Line 76; pp 3]**  h. differential list was referring to the WNV was not included in one of the differential diagnosis lists. **[Line 87; pp 4]**  i.The figure (Supplementary Figure 1) has been deleted as suggested by the reviewer accordingly.  j. The entomologist was mentioned in acknowledgement section. **[Line 367; pp 18 ]**  k. The author apologizes for the confusion. The mosquitoes were identified up to species level and the sentences was amended accordingly **[Table 2, pp14]**  l. The study of JEV RNA on mosquitoes was carried out by another student in our group, which all of the mosquitoes are negative for JEV RNA [unpublished data] [**Line 179; pp:8**]  m. The primer was blasted for validation (https://blast.ncbi.nlm.nih.gov/Blast.cgi) which successfully shown detection on both conserved region from Lineage 1 and 2 of WNV **[Line 186; pp 8 ]**  n. The figure (supplementary figure 2) has been deleted as suggested by the reviewer accordingly.  o. The sentences and the associated figure has been deleted as suggested by the reviewer accordingly.  p. The author apologizes for the confusion. The mosquitoes were identified up to species level and the sentences was amended accordingly **[Line 160; pp 7]**  q. The word has been amended as suggested by the reviewer. **[Line 257; pp:11]**  r. The positive control was designed with the alignment of 17 WNV nucleotide sequences as stated in previous study by Ain-Najwa et al. (2020) which contain conserved region of Lineage 1 and Lineage 2. **[Line 266; pp:12]**  s. Table 2 has been labeled accordingly. **[Line 293; pp:14]**  t. The discussion has been amended by adding only principle findings of this study. **[Line 299; pp:16]**  u. The sentence has been amended accordingly **[Line 301; pp:16]**  v. The correct similarity was added **[Line 334; pp 17]** |
|  | Reviewer 3 | - | - |
|  | Reviewer 4 | a. In lines 137-138, the authors don’t sound convincing on the suitability of the sites  b. The population is clearly described and appropriate for the methods being described, but the challenge is still the absence of a testable hypothesis  c. There is no mention or calculation for an ideal/desired/minimum sample size of mosquitoes to be collected.  d. From line 251 and Table 3, it’s not clear why the number of pools and number of mosquitoes per pool was chosen. (RESULT)  e. The generally expected regulatory requirements seem to have been met but it’s unclear from the methods section if ethical approval was sought from an institutional review board. If this were not necessary, then an explanation of the grounds for an exemption would have sufficed.  f. The statistical analysis methods and results support the conclusions. However, it is difficult to assess the appropriateness of the conclusions because the objectives of the study were not clearly stated. | ***The authors would like to thank the reviewer for the comment.***    a. The sentences has been amended accordingly **[Line 137; pp 6]**  b. The hypothesis has been added as suggested by the reviewer **[Line 221; pp 10]**  c. Convenient sampling method was chosen in this study at which sample size greater than 30 and less than 500 are appropriate for most research as mentioned by Roscoe (1975) **[Line 134; pp 6]**  d. It was chosen those way based on the availability of the trapped mosquito species and to effectively calculate the MIR of WNV. The authors believed that standardization of the number of pools is the best and ideal, however factors such as species and sex were taken into consideration with maximum pooling of 10 mosquitoes. **[Table 2, pp14]**  e. The ethical statement already been included in the methodology section **[Line 109; pp 5]**  f. The objective of this study was stated in the introduction part, and we have added the hypothesis of this study as well **[Line 103; pp 5]** |
|  | Reviewer 5 | - The purpose/objectives of this study are clearly understood and the methods applied are adequate to test the hypothesis of WNV being present in Malaysian mosquitoes that live near migratory bird roosting sites.  The mosquito population tested is limited to a small window, but the snapshot style of surveillance does provide a starting point for further investigation and can be used to drum up interest in the subject. The population is able to address the hypothesis tested. The statistical analysis performed fits the data tested, and supports the conclusions drawn by the authors.  a. Line 165: The description of the pools of mosquitoes is lacking. In the Results section (line 258) the authors state that only one pool from 2017 was positive and the remaining positive pools were from 2018.  However, the number of total pools from each year is not apparent.  This impacts the readers understanding of the situation, as if only 3 pools were collected in 2017, this would mean that 33% pools in 2017 were positive compared to in 2018 where 12% were positive.  Whereas, if the pools are evenly split between years, 0.7% (1/142) in 2017 were positive compared to 23.7% (34/143) in 2018.  Furthermore, more description could have been provided by stating how many pools were collected at each site for each year.  This would have provided an even clearer picture  b. Line 181: In the description of the Nested RT-PCR the master mix manufacturer used for the PCR is not listed.  c. Line 200: The authors state that the RT step of RT-PCR occurred at 95 °C for 20 minutes.  I believe this to be a typo as the RT step generally occurs at between 45 and 55 °C and 95 °C is used in the next step (polymerase activation) so as written no change in temperature occurred between these two steps, thus there was no end of the RT step/beginning of the polymerase step. | ***The authors would like to express great appreciation for the reviews and comments***  a. Considering that the sample was only captured within the particular time frame, we have omitted this part as month/year was not one of our variables during the sampling [**Line 159; pp:7]**  b. The manufacturer information has been added accordingly **[Line 178; pp 8]**  c. The temperature has been amended to the correct temperature **[Line 194; pp 8]** |
| Result | Reviewer 1 | a. Table 3 are the heart of the paper as was Figure 2 showing virus phylogeny.   Again, it was unclear how species specific infection rates were determined when specimens were 'pooled according to genus'?  This makes calculation of species specific MIRs difficult to understand.  In addition, because there were variable numbers of specimens within pools, it would have been better to calculate MLEs which account for variable numbers of specimens tested per pool.  b. WNV RNA detected in mosquito pools are not really isolates.  Isolates are typically whole viruses preserved in culture or by ultralow freezing | ***The authors would like to thank the reviewer for the comment***.    a. The mosquitoes were identified up to species level and the sentences was amended accordingly. **[Table 2, pp 14]**  According to Gu et al., 2003, when having inconsistent pool size, MLE is a not suitable indicator to be used to estimate infection rate. [https://doi.org/10.1603/0022-2585-40.5.595]. Therefore, we would like to remain the MIR, which was also had been suggested by the other two reviewers. **[Line 215; pp 9]**  b. The “isolate” term has been deleted accordingly. |
|  | Reviewer 3 | - | - |
|  | Reviewer 4 | The analysis presented is in line with the title and the background information, but it's unclear what the analysis plan was from the onset. Parts of the discussion contain data that should ordinarily be found in the results section. For instance, lines 350-352. The results are clearly presented. The completeness of the results would be judged better in the presence of a testable hypothesis. The figures are of sufficient quality for clarity. | ***The authors would like to thank the reviewer for the comment.***  The part has been deleted and hypothesis has been added accordingly |
|  | Reviewer 5 | The authors clearly delineate and address the results from each piece of their analysis plan in the results section and arrange the sections in a manner that follows a logical pattern.  Each of the figures was of high enough quality to clearly see the results expressed.  a. Line 230 – 248: The mosquito identification section is a bit longer than needed much of the information could have been better summarized in a supplementary table, with examples given in the text.  b. Line 236: The sentence states that 7 species of the Culex genus were identified, then lists the species specifically.  However, the final listed species, simply says Culex spp.  Why is this not further elucidated? | ***The authors would like to express great appreciation for the reviews and comments***  a. The part has been amended accordingly. **[Line 231; pp 10]**    b. The species of mosquito cannot be further identified due to damage on required morphological features of specimen **[Line 296; pp 15]** |
| Conclusion | Reviewer 1 | Conclusions were appropriate | - |
|  | Reviewer 3 | Partially, but I have highlighted the necessary modifications in order to fulfill these requirements in the "Summary and General comments" box. | - |
|  | Reviewer 4 | The first two paragraphs of the Discussion section sound more like background information rather than data supporting (discussing) the findings. The larger part of the conclusions is supported by the results presented. The study/ analysis limitations are omitted. The discussion on the importance of the data is satisfactory. This includes the public health relevance of the findings. | ***The authors would like to express great appreciation for the reviews and comments***  The discussion has been amended as suggested by the reviewer **[Line 299; pp 16]** |
|  | Reviewer 5 | In the discussion the authors appropriately address the limitations of this study, explaining why the methods they used, which admittedly selected for certain mosquito species.  The authors do a good job of not over stating the findings of their study.  The length of the discussion is a bit long, as they do restate much of what is said in the results, which could be rectified by referencing the results section and briefly summarizing what was written there.  They also refer back to the introduction in order to address the public health implications.  The authors also discuss how the results of this investigation may lead to further surveillance which I believe was the ultimate goal of the study, as the purpose introduced earlier was to discover if WNV was under surveilled and is more prevalent in Malaysia than previously known.  a. Line 352: The authors state that *Culex tritaeniorhynchus* had the highest MIR, when technically other species did. Understandably, the authors did not assume that the 1/1 WNV positive *Coquillettidia* was a realistic MIR, however, this technicality should be addressed, as well as the authors’ reasoning as to why they censored this percentage, and how they addresses the low numbers of several other species.  b. Lines 386-395: The authors discuss the environmental conditions of the study sites, but do not discuss the specifics conditions observed.  It would be helpful to have conditions laid out, such as cumulated rainfall over the past weeks, and average temperatures (temperature is stated in the study site description in the method, but only the temperature at the time of collection).  The authors continue on to reference an article in which temperature affects WNV transmission, but do not discuss how this may have impacted the results of their study.  c. Line 309-314: This section could use further clarification, as upon first read the meanings were not explicit. | ***The authors would like to thank the reviewer for the comment.***  a.The authors agree with the reviewer opinion about the other species like *Coquillettidia* spp. when had 1/1 WNV positive, was a realistic MIR. Since the trapping method (CDC light trap) influences the species of the mosquito collected, it explains why we acquired low number of other species of mosquito. We had added this information in the discussion [**Line 326; pp 17]**  b. We apologized that the information of the cumulated rainfalls and average temperature were no longer available in the meteorological department. However, during sampling, both sites experienced monsoon season weather patterns whereby the rainfall was higher than normal period coupled with thunderstorms as alerted by the official website of Malaysian Meteorological Department. **[Line 138-140; pp 6]**  c. The sentence has been amended as suggested by the reviewer **[Line 285; pp 13]** |
| Editorial and Data Presentation Modifications | Reviewer 1 | - The paper is too long for the limited amount of sampling done - two traps on two sampling occasions at two locations.   It contains too much general information and extraneous details.  I have edited the attached file trying to show what areas could be removed/condensed.   The data confirms that mosquitoes collected in Malaysia during bird migration were infected with WNV -- period.  Species specific data is useful if the pools indeed contained individual species of Culex. | ***The authors would like to express great appreciation for the revision and editing of the manuscript.***  The comments were taken into account seriously and amended accordingly. |
|  | Reviewer 3 | - | - |
|  | Reviewer 4 | Minor revision recommended. To include clear objectives, hypotheses, and limitations. | ***The authors would like to express great appreciation for the revision and editing of the manuscript.***  Clear hypothesis, objectives and limitations have been added/amended throughout the manuscript as suggested by the reviewer. |
|  | Reviewer 5 | - | - |
