# Supplementary material for: Circulation of West Nile virus in mosquitoes approximate to the migratory bird stopover in West Coast Malaysia
Source: PLoS Negl Trop Dis. 2023 Apr 6;17(4):e0011255. doi: 10.1371/journal.pntd.0011255 (PMC10112790; doi:10.1371/journal.pntd.0011255)
Supplement: Attachment — Submitted filename: Rebuttal Letter and Response to Reviewer Comments.docx [file pntd.0011255.s008.docx]

21^st^ March 2023

Editor-in-Chief,

PLOS Neglected Tropical Disease,

Shaden Kamhawi,

Vector Molecular Biology,

National Institutes of Health,

Rockville, MD, USA

Dear Editor,

REVISED MANUSCRIPT FOR PUBLICATION

Enclosed herewith is a revised manuscript entitled “Circulation of West Nile Virus in Mosquitoes Approximate to the Migratory Bird Stopover in West Coast Malaysia” for publication in PLOS Neglected Tropical Disease.

We thank reviewers for suggestions and comments. We the feel that the reviewer's comments helped clarify and improve the manuscript. The response to the comments is attached. With the submission of this manuscript, I would like to undertake that the above-mentioned manuscript has not been published elsewhere, accepted for publication elsewhere, or under editorial review for publication elsewhere.

I hope the manuscript is in satisfactory condition and has the merit for publication in PLOS Neglected Tropical Disease.

Thank you in advance.

With best regards,

------------------------------------------------

Dr. Nor Yasmin Abd. Rahaman

Corresponding author

E-mail: [noryasmin@upm.edu.my](mailto:noryasmin@upm.edu.my)

**Response to Reviewers**

Dear Reviewers,

The authors have revised the manuscript accordingly and feel that the reviewer's comments helped clarify and improve the manuscript. Kindly please find our responses.

| **Section** | **Reviewer** | **Comments** | **Response** |
| --- | --- | --- | --- |
| **Method** | Reviewer 1 | The purpose of the study was clear | ***The authors would like to thank the reviewer for the comment.*** |
|  | Reviewer 5 | Introduction: Line 105 - Hypothesis statement has some grammatical issues and reads as if the hypothesis is that wild birds are the bloodmeal source for WNV infected mosquitoes in the sample areas. Given that the paper does not test this, the statement should be reworded to hypothesize the presence of WNV in the mosquitoes of these areas, as that was what was tested. | ***The authors would like to express great appreciation for the reviews and comments. The comments were taken into account seriously and amended accordingly.***  The sentence has been amended accordingly. **[Line 103; pp: 5]** |
| **Results** | Reviewer 1 | The Results are clearly presented. The authors should carefully recheck their MIR calculations per species? I got different results [see attached manuscript]. | ***The authors would like to thank the reviewer for the comment. The author apologizes for the confusion. The comments were taken into account seriously and amended accordingly.***  According to Bernard et al., (2001), MIR can be calculated only if a minimum of 1,000 mosquito were collected. Since the total mosquito in this study was 2635, the overall MIR value is included. However, the calculation of the MIR per species of mosquito was not proceeded as the total number of mosquitoes per species is not exceeding 1000 mosquitoes. Thus, the value of MIR per species were omitted.  Reference: [10.3201/eid0704.010415](https://doi.org/10.3201/eid0704.010415)  **[Line 207, 269; pp: 9, 12]** |
|  | Reviewer 5 | Line 288: Minor grammatical issue makes sentence harder to read | ***The authors would like to express great appreciation for the reviews and comments. The comments were taken into account seriously and amended accordingly.***  The sentence has been amended accordingly. **[Line 271; pp: 11]** |
| **Conclusion** | Reviewer 1 | The conclusions are supported by the data. | ***The authors would like to thank the reviewer for the comment.*** |
|  | Reviewer 5 | No response | ***-*** |
| **Editorial and Data Presentation Modifications** | Reviewer 1 | I downloaded the .pdf version and from this downloaded the cleaned .docx version. I read this version and added edits/comments to improve English, clarity and syntax. Hopefully this will allow the authors to prepare an acceptable final version.  The authors use the terms virus, infection and disease in the same context, which I feel is incorrect. The name of virus should be used when discussing the pathogen, infection occurs after the pathogen is transmitted to the host, and disease describes the response of the host to the infection. | ***The authors would like to express great appreciation for the reviews and comments. The comments were taken into account seriously and amended accordingly.*** |
|  |  | ***[from track changes*]**  Viruses emerge not infections. **[Line 75; pp: 3]** | ***[from track changes*]**  The sentence has been amended accordingly. **[Line 75; pp: 3]** |
|  |  | Unclear: As humans are a dead end host for the virus how does human infection have zoonotic implications? **[Line 75; pp: 3]** | The sentences has been rephrase as suggested by the reviewer **[Line 75; pp: 3]** |
|  |  | Unclear: what is this list? Who has put it together **[Line 86; pp: 4]** | The sentence has been amended. Other mosquito borne suspected diseases such as dengue or chikungunya virus infection contributes similar clinical manifestation. **[Line 83; pp: 4]** |
|  |  | Unclear: 2 traps in convenient locations? **[Line 115; pp: 5]** | Yes, two traps were installed in these sites as positive WNV on birds were reported in these sites previously. **[Line 116; pp: 5]** |
|  |  | Unclear **[Line: 190; pp: 8]** | The sentence has been amended as suggested by the reviewer **[Line: 191; pp: 8]** |
|  |  | Denominator is the total mosquitoes tested in both positive and negative pools? **[Line: 207; pp: 9]** | The denominator is the total mosquitoes tested both positive and negative, which is 2635 mosquitoes including female and male mosquito. Male mosquito was added in the calculation as WNV could be transmitted vertically in mosquito population **[Line: 209; pp: 9]** |
|  |  | Recheck the MIR calculations **[Line 269; pp: 12]** | Thank you for the comment. The MIR value and the discussion was amended accordingly **[Line 269, 295; pp: 12, 15]** |
|  | Reviewer 5 | No response | ***-*** |
| **Summary and General Comments** | Reviewer 1 | The paper should be acceptable after one more careful revision | ***The authors would like to thank the reviewer for the comment.*** |
|  | Reviewer 5 | No response | ***-*** |
